# Whole-genome resequencing of 472 *Vitis* accessions for grapevine diversity and demographic history analyses

**DOI:** 10.1038/s41467-019-09135-8

**Published:** 2019-03-13

**Authors:** Zhenchang Liang, Shengchang Duan, Jun Sheng, Shusheng Zhu, Xuemei Ni, Jianhui Shao, Chonghuai Liu, Peter Nick, Fei Du, Peige Fan, Ruzhi Mao, Yifan Zhu, Weiping Deng, Min Yang, Huichuan Huang, Yixiang Liu, Yiqing Ding, Xianju Liu, Jianfu Jiang, Youyong Zhu, Shaohua Li, Xiahong He, Wei Chen, Yang Dong

**Affiliations:** 1 Beijing Key Laboratory of Grape Sciences and Enology, Laboratory of Plant Resources, Institute of Botany, Chinese Academy of Sciences, Beijing, 100093 China; 2 Sino-Africa Joint Research Center, Chinese Academy of Sciences, Wuhan, 430074 China; 3 State Key Laboratory for Conservation and Utilization of Bio-Resources in Yunnan, Yunnan Agricultural University, Kunming, 650201 China; 4 Nowbio Biotechnology Company, Kunming, 650201 China; 5 Yunnan Research Institute for Local Plateau Agriculture and Industry, Kunming, 650201 China; 6 Key Laboratory for Agro-biodiversity and Pest Control of Ministry of Education, Yunnan Agricultural University, Kunming, 650201 China; 7 BGI, BGI-Shenzhen, Shenzhen, 518120 China; 8 BGI Institute of Applied Agriculture, BGI-Shenzhen, Shenzhen, 518120 China; 9 Zhengzhou Fruit Research Institute, Chinese Academy of Agricultural Sciences, Zhengzhou, 450009 China; 10 Botanical Institute, Karlsruhe Institute of Technology, Karlsruhe, 76128 Germany; 11 University of Chinese Academy of Sciences, Beijing, 100049 China

## Abstract

Understanding the *Vitis* species at the genomic level is important for cultivar improvement of grapevine. Here we report whole-genome genetic variation at single-base resolution of 472 *Vitis* accessions, which cover 48 out of 60 extant *Vitis* species from a wide geographic distribution. The variation helps to identify a recent dramatic expansion and contraction of effective population size in the domesticated grapevines and that cultivars from the pan-Black Sea region have a unique demographic history in comparison to the other domesticated cultivars. We also find selective sweeps for berry edibility and stress resistance improvement. Furthermore, we find associations between candidate genes and important agronomic traits, such as berry shape and aromatic compounds. These results demonstrate resource value of the resequencing data for illuminating the evolutionary biology of *Vitis* species and providing targets for grapevine genetic improvement.

## Abstract (web summary)

Despite the importance of grapevine cultivation in human history and the economic values of cultivar improvement, large-scale genomic variation data are lacking. Here the authors resequence 472 *Vitis* accessions and use the identified genetic variations for domestication history, demography, and GWAS analyses.

## Introduction

Domesticated grapevine (*Vitis vinifera* ssp. *vinifera*) is the most cultivated fruit crop in the genus *Vitis*, which also contains about 60 inter-fertile wild species^1–3^. Within their native habitat in the temperate regions of the world, there are about 28 wild *Vitis* species indigenous to North America and about 30 wild *Vitis* species indigenous to East Asia^1–3^. *V. vinifera* ssp*. sylvestris* is the only extant wild *Vitis* taxon native to Europe and Near East, and it is believed to be the wild progenitor for almost 10,000 domesticated grapevine cultivars today^1–3^. In addition, about 1000 commercial grapevine cultivars are interspecific hybrids of the domesticated grapevines and other wild *Vitis* species^2^. The berries of the grapevine plants are either consumed directly as fresh fruit and raisins or made into various alcoholic and nonalcoholic beverages. In the context of cultural and religious exchange, the agricultural exploitation of grapevines has greatly influenced the human race ever since civilization flourished in the Near East.

Despite the importance of grapevine cultivation in human history and the economic values of cultivar improvement, large-scale genomic variation data for grapevines are lacking. Besides the widely used 10–20 K genotyping arrays^4,5^, the whole-genome resequencing data of various qualities were only reported very recently for 36 grapevine accessions in total^6–8^. The deficit of genomic resource has hampered the investigation of the past history and the present trait improvement of grapevines. For example, current knowledge based on archeological excavation and chemical analysis of the wine potteries in Georgia argued for the earliest viniculture in the Near East at about 8000 years ago^9^. This domestication origin for grapevine was supported by a genotyping analysis of 1000 accessions^10^, and the distribution of chlorotype variations of 1200 grapevines suggested an additional area of origin in the western Mediterranean^11^. However, the domestication time and the demographic history of grapevine based on genomic data remain elusive. Secondly, the biology of grapevine went through significant changes during domestication, with the most noticeable ones being seed shape and flower sex^3^. Without large-scale variation data, it is impossible to discern genomic regions under selective sweep on a global scale that might give rise to important domestication traits. Lastly, the majority of the domesticated grapevine cultivars are susceptible to disease. At the end of the 19th century, mildews and Phylloxera pests from North America devastated the vineyards and wild grapevines in Europe^3^. For this reason, a sustainable viticulture will rely on harnessing the genetic diversity of not only grapevines in cultivation, but also *Vitis* species in the wild^10^.

With these points in mind, we propose to delineate the whole-genome genetic variations at single-base resolution from 472 *Vitis* accessions and four closely-related species in other genera of the Vitaceae family from a wide geographic distribution (Supplementary Data 1). These accessions include 64 wild European accessions (*V. vinifera* ssp*. sylvestris*, WEU), 44 accessions from 26 wild East Asian species (WEA), 35 accessions from 21 wild North American species (WNA), 220 accessions from 177 domesticated grapevine cultivars (*V. vinifera* ssp. *vinifera*, CEU), and 109 accessions from 69 interspecific-hybrid grapevine cultivars (HYB). Considering that there are about 11,000 acknowledged grapevine cultivars and 60 *Vitis* species, this resequencing collection as an effort of three institutions aims to capture as many genetic variations in the *Vitis* species as possible, provide the largest resource to date to facilitate the breeding of new grapevine cultivars, and serve as a stepping stone for more extensive collaborations in future to investigate grapevine genetic diversity. With the identified single-nucleotide polymorphisms (SNPs), we find a recent dramatic expansion and contraction of effective population size in the domesticated grapevines and a unique demographic history in cultivars from the pan-Black Sea region. We also find selective sweeps for berry edibility and stress resistance improvement. Furthermore, we find associations between candidate genes and important agronomic traits, such as berry shape and aromatic compounds.

## Results

### SNPs and genomic structural variations

About 4.1 Tb of whole-genome sequencing data (27.3 billion paired-end raw reads) were generated for 472 *Vitis* accessions and four other closely-related species at an average depth of ~15.5× (Supplementary Fig. 1). The mapping rate of these raw reads to the *V. vinifera* reference genome^12^ was 97.4 ± 4.6%, and the estimated error rate was 0.02 ± 0.01% (Supplementary Data 2). Additionally, the genome coverage was more than 80% across all chromosomes for the majority of accessions (Supplementary Fig. 2).

In order to assess the genetic diversity in both wild and cultivated *Vitis* accessions, we mapped all individuals to the Pinot Noir reference genome^12^, a method also used in the analyses of large resequencing datasets of other plant genera, such as *Malus*^13^, *Citrus*^14^, and *Cajanus*^15^. After applying basic filtering criteria (see Methods) we identified 77,726,929 SNPs, 10,278,017 short genomic insertions and deletions (indels), and about 25,000 copy number variants. Further filtering yielded a basic set of 37,859,960 SNPs and 3,854,659 indels (≤40 bp) with minor allele frequency (MAF) more than 0.005, and a core set of 12,549,273 SNPs and 904,280 indels (≤40 bp) with MAF more than 0.05. The ratios of transition to transversion (Ti/Tv) SNPs for the basic set and core set were estimated to be 2.48 and 2.88, respectively, showing the high quality of the SNP call sets.

**Fig. 1:**
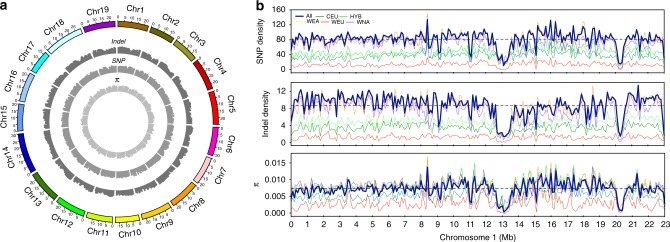
Summary of the genomic variations identified across 472 *Vitis* accessions. **a** Circos graph representation of the SNP density, indel density, and total genetic diversity (*π*) across all chromosomes of the grapevine genome scaled to Mb size. **b** Panoramas of the SNP density, indel density, and total genetic diversity across chromosome 1 of the grapevine genome with contiguous 100 kb subregions. The dashed line represents the average values for the entire genome. WNA (purple), WEA (yellow), WEU (red), CEU (dark green), and HYB (light green) represent wild North American *Vitis* species, wild East Asian *Vitis* species, wild European grapevine, domesticated grapevine cultivars, and interspecific-hybrid grapevine cultivars, respectively. This color scheme is used throughout the manuscript

A survey of all the identified indels and SNPs in the grapevine genome showed that every chromosome contained regions of high indel and SNP density that deviated from the whole genome averages (Fig. 1). About 71.2% of SNPs were located in the intergenic regions and 4.6% in the coding sequences. The nonsynonymous-to-synonymous substitution ratio for the SNPs in the coding regions was 1.17, which is comparable to the values reported for pigeonpea (1.18)^15^, but lower than the values reported for tomato (1.23)^16^, soybean (1.35)^17^, and rice (1.46)^18^. We also found that 73.6% of indels were located in the intergenic regions and 1.4% in the coding regions. An estimated 66.7% of indels in the coding regions could cause frameshift mutations.

### Analysis of *Vitis* phylogeny

**Fig. 2:**
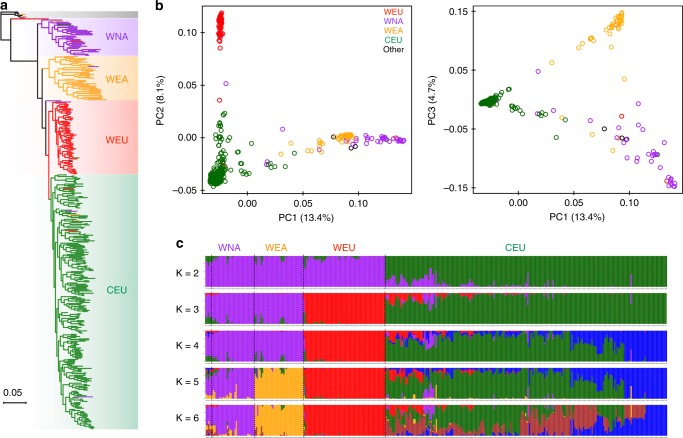
Phylogeny and population structure of major *Vitis* categories. **a** Maximum likelihood phylogenetic tree of grapevine accessions inferred from the whole-genome SNPs with 100 nonparametric bootstraps (see Supplementary Fig. 3 and 4 for bootstrap values as blue circles). *Parthenocissus tricuspidata* (Boston ivy) was used as an outgroup. **b** PCA plots of the first three components of major *Vitis* accessions using whole-genome SNP data. **c** Population structure of major *Vitis* categories estimated by ADMIXTURE. Each color represents one ancestral population. Each accession is represented by a bar, and the length of each colored segment in the bar represents the proportion contributed by that ancestral population

The core set of SNPs were used to analyze the phylogeny and population structure of the *Vitis* accessions. Maximum likelihood (ML) phylogenetic analysis with 100 nonparametric bootstraps revealed distinct monophyletic clades for wild North American *Vitis* species (WNA, purple), wild East Asian *Vitis* species (WEA, yellow), wild European species (WEU, red), and domesticated grapevine cultivars (CEU, dark green, Fig. 2a and Supplementary Fig. 3). This topology supports previous reports that the *Vitis* clade containing wild North American species (New World species) is sister to the Eurasian *Vitis* clade containing wild East Asian, wild European, and domesticated grapevine species^19,20^. Within the Eurasian *Vitis* clade, wild European *Vitis* is a sister clade to domesticated grapevine species, which in turn, as a whole clade, is sister to wild East Asian *Vitis* clade. A closer examination of the phylogenetic nodes (bootstrap value ≥ 75) showed that at least two and five smaller groups existed in the wild North American and wild East Asian *Vitis* clades, respectively (Supplementary Fig. 3). The majority of interspecific-hybrid grapevine cultivars (HYB, light green) were classified into two separate clusters (Supplementary Fig. 4), mainly reflecting their various hybridization background among wild North American, wild East Asian, and domesticated grapevine cultivars. The ML phylogenetic tree also revealed a few accessions being grouped into other *Vitis* clades of different genetic background, suggesting potential misclassification of accessions based on morphology during sample collection^21^. For instance, TA-5901 from St. Andres of Canary Islands was originally believed to be *V. vinifera*. The phylogenetic tree suggested that this tree-like specimen might well be a *V. sylvestris* from Northern Africa. Another example is TA-6147, which was identified as a *V. sylvestris* in the UC Davis collection (introduced to KIT in 2006). A previous study using plastid markers cast doubt on this taxonomy as it clustered with wild North American species^22^. Our phylogenetic tree confirmed this misidentification, and showed that TA-6147 might be a *Vitis acerifolia* sample. A complete description of these misidentifications is in Supplementary Data 1.

### Analysis of *Vitis* population structure

Principal component analysis (PCA) showed substantial genetic diversity among major grapevine categories, with the first three principal components explaining 13.4%, 8.1%, and 4.7% of the total genetic variance, respectively (Fig. 2b). PC1 separated Eurasian accessions (WEU and CEU) from wild East Asian and wild North American accessions, suggesting that the latter two grapevine categories shared more similarity in the genetic background than Eurasian accessions. This finding is further supported by the result of model-based analyses of population admixture (Fig. 2c, *K* = 3; and Supplementary Fig. 5a, c). PC2 evidently separated wild European accessions from domesticated grapevine accessions, whereas PC3 set wild North American accessions apart from wild East Asian accessions (Fig. 2b). The differentiation between these major grapevine categories was also evident in the population admixture graph (Fig. 2c, *K* = 5; and Supplementary Fig. 5a and 5c). Additional PCA analysis showed that the interspecific-hybrid grapevine cultivars were strewn in between other grapevine categories (Supplementary Fig. 5b). Their relative positions in the graph reflected the level of genomic influence from each parent *Vitis* species. In the admixture plot at *K* = 4 (Supplementary Fig. 5c), it is apparent that the majority of interspecific-hybrid grapevine cluster 1 (HYB1) received genetic contributions from wild North American, wild East Asian, and domesticated grapevine species, whereas the interspecific-hybrid grapevine cluster 2 (HYB2) received genetic contributions from only wild North American and domesticated grapevine species. This result is in line with the ML phylogenetic tree (Supplementary Fig. 4). Even though most of the domesticated grapevine accessions were closely clustered in the PCA graphs, they showed a clear pattern of high genetic heterogeneity as evidenced by the population admixture analyses (Fig. 2c, *K* = 6; and Supplementary Fig. 5c, *K* = 8).

### Analysis of linkage disequilibrium

**Fig. 3:**
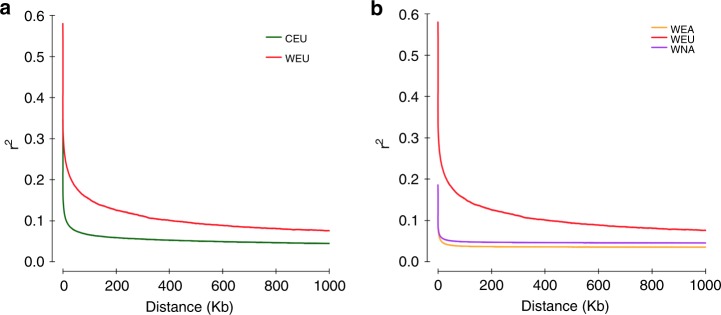
Decay of linkage disequilibrium of major *Vitis* categories. **a** Decay of linkage disequilibrium in grapevine genome for wild European grapevines and domesticated grapevine cultivars. **b** Decay of linkage disequilibrium in grapevine genome for extant wild *Vitis* species

Characterization of the linkage disequilibrium (LD, expressed as *r*^2^) pattern is crucial to forward genetics studies in plant^23^. Previous evaluations of LD decay in wild European and domesticated grapevine cultivars using small-scale genetic markers yielded inconsistent results, with some studies showing a relatively small LD extents at 10–20 kb^10,24^, whereas the others showing a large LD extents at 28–458 kb^5,25^. With the whole-genome SNPs available, we found that the decay of LD reached half of maximum average *r*^2^ at a distance of 2.9 kb for wild European grapevines and 350 bp for domesticated grapevines (Fig. 3a). These parameters are substantially smaller than previous reports and those found in wild soybean (~27 kb)^17^, wild rice (20 kb)^26^, and wild maize (22 kb)^27^. The relatively slower decay of LD in wild European species versus domesticated grapevine cultivars is concordant with previous findings^24^, but it is important to realize that this difference may narrow with a more diverse wild European *Vitis* population. In comparison to wild European species, we also found that LD decayed rapidly to an average *r*^2^ of 0.1 within 300 bp for both wild North American and wild East Asian *Vitis* species (Fig. 3b).

### Demographic histories of WEU and CEU grapevines

**Fig. 4:**
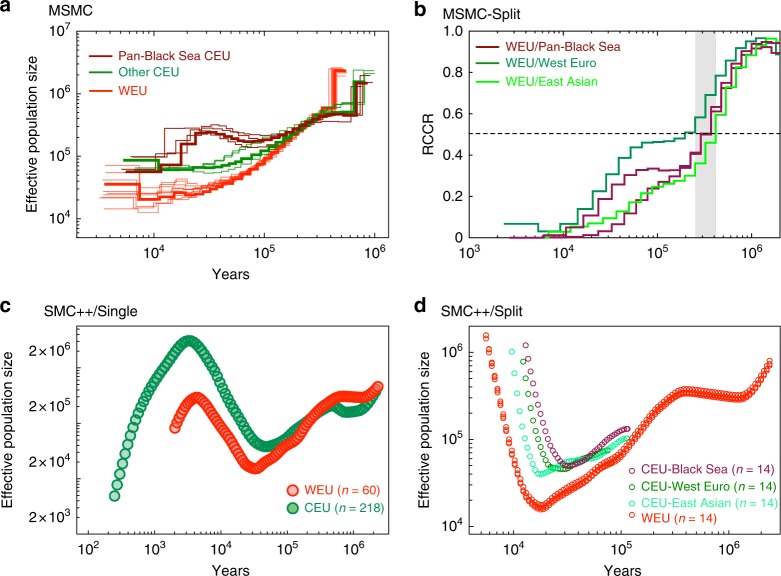
Demographic history of wild European grapevines and cultivated grapevines. **a** MSMC-derived demographic history of WEU and CEU grapevines from 10^4^ to 10^6^ years ago. Each line represents a run of four haplotypes from two individual accessions. An example was highlighted in each group. For CEU grapevines, cultivars from the pan-Black Sea region generated distinct demographic history compared with other cultivars. **b** MSMC split analysis of WEU and various subgroups of CEU grapevines based on relative cross-coalescence rate (RCCR). **c** Demographic history of WEU (*n* = 60) and CEU grapevines (*n* = 218) from 10^2^ to 10^6^ years ago from SMC++ single population analysis. **d** Demographic history of WEU (*n* = 14) and various subgroups of CEU (*n* = 14) grapevines from 10^4^ to 10^6^ years ago from SMC++ split analysis

The demographic history of the domesticated annual crops, such as maize^28,29^ and African rice^30,31^, is characterized by a sequential contraction and expansion of estimated effective population size (*N*_*e*_) around the time of domestication. However, this pattern (particularly *N*_*e*_ expansion) was not observed in the domesticated grapevine, a perennial crop with the capability of vegetative propagation^6^. To revisit this question, we applied the multiple sequentially Markovian coalescent (MSMC)^32^ model to the analysis of phased SNP data from both wild European grapevines and domesticated grapevines (Fig. 4a). The results were scaled to real time by assuming a generation time of 3 years^10^ and a neutral mutation rate of 5.4 × 10^−9^ per year (see Methods). As shown in Fig. 4a, wild European grapevines (red lines) experienced a steady decline of *N*_*e*_ from the highest point (*N*_*e*_ ≈ 600,000) at 400 Kya to the nadir (*N*_*e*_ ≈ 20,000) at 10 Kya. Similar demographic patterns were observed for the majority of the domesticated grapevines in the world (green lines), with the lowest *N*_*e*_ ≈ 60,000 around 10–20 Kya. Interestingly, domesticated grapevines from the pan-Black Sea region (maroon lines, Caucasus region and west coast of Black Sea) manifested a unique mild *N*_*e*_ expansion (*N*_*e*_ ≈ 150,000 up to *N*_*e*_ ≈ 300,000) around 30–70 Kya and a subsequent moderate *N*_*e*_ contraction (*N*_*e*_ ≈ 300,000 down to *N*_*e*_ ≈ 60,000) around 10–30 Kya. Figure 4a also shows that the population of all domesticated grapevines started to differ from wild European grapevines at a similar time around 250–300 Kya. From analyzing eight haplotypes for pairs of populations in the MSMC split analyses (Fig. 4b), we found that the relative cross coalescence rate reached 0.5, suggesting the predicted split state, at around 250–400 Kya between wild European grapevines and domesticated grapevines (all lines).

After population divergence, the change in the relative cross-coalescence rates for the pan-Black Sea cultivars followed two unique patterns (maroon lines): one group resembled the Western European cultivars (dark green line) and the other group resembled the East Asian cultivars (light green line; Fig. 4b).

Since the power of MSMC is quite limited for predictions more recent than 10 Kya in this study, we explored the recent demographic history of grapevines with unphased SNP data using SMC++^33^. Single population approach revealed that domesticated grapevines (*n* = 218) experienced a prominent *N*_*e*_ expansion (Fig. 4c; *N*_*e*_ ≈ 40,000 up to *N*_*e*_ ≈ 3,000,000) around 3000 ya to 40 Kya and a subsequent severe *N*_*e*_ contraction (down to *N*_*e*_ ≈ 5000) around 400–3000 ya. The wild European grapevines experienced *N*_*e*_ expansion and contraction during similar period of time (Fig. 4c; *N*_*e*_ ≈ 15,000 up to *N*_*e*_ ≈ 300,000, then down to *N*_*e*_ ≈ 80,000). The split analyses using domesticated grapevines of various geographical regions (Fig. 4d) showed that the divergence times of pan-Black Sea, Western European, and East Asian cultivars from wild European grapevines were at about 80–100 Kya. The pan-Black Sea and East Asian cultivars also seemed to diverge from the wild European grapevines earlier than Western European cultivars. The *N*_*e*_ expansion started earlier for the pan-Black Sea cultivars (~30 Kya) than for Western European (~22 Kya) and East Asian cultivars (~18 Kya). Due to the small sample size of the available pan-Black Sea cultivars, the ensuing *N*_*e*_ contractions for these subgroups of grapevines could not be inferred (Fig. 4d). Taking both MSMC and SMC++ results together, we propose that the pan-Black Sea cultivars underwent a unique demographic history ever since their divergence from the wild European grapevines. In addition, all grapevines may have undergone at least one bottleneck since the Last Ice Age (see Discussion). Due to the difficulty in identifying a true progenitor population in grapevine research, we advise all analyses involving the wild European grapevines be interpreted with caution.

### Pedigree analysis within *Vitis* accessions

The accurate reconstruction of grapevine genealogy from genomic data is difficult due to the coexistence of vegetative propagation and sexual reproduction^10^. Nevertheless, we analyzed the patterns of identity-by-descent (IBD) relationships among the 472 *Vitis* accessions. The histogram of IBD values from pairwise *Vitis* comparisons is bimodal (Supplementary Fig. 6a). No pairwise IBD values exceeded 0.95, the empirical cut-off for defining clonality^10^. By defining a cut-off IBD value of 0.420 (lowest value that separates two modes), we found that 335 *Vitis* accessions (71.0%) were related to at least one other accession by a first-degree relationship. When the lowest pairwise IBD value (0.466) for 43 confirmed *Vitis* parent–offspring pairs was used^10^, 292 *Vitis* accessions (61.9%) retained a first-degree relationship with at least one other accession (Supplementary Fig. 6b, c).

**Fig. 5:**
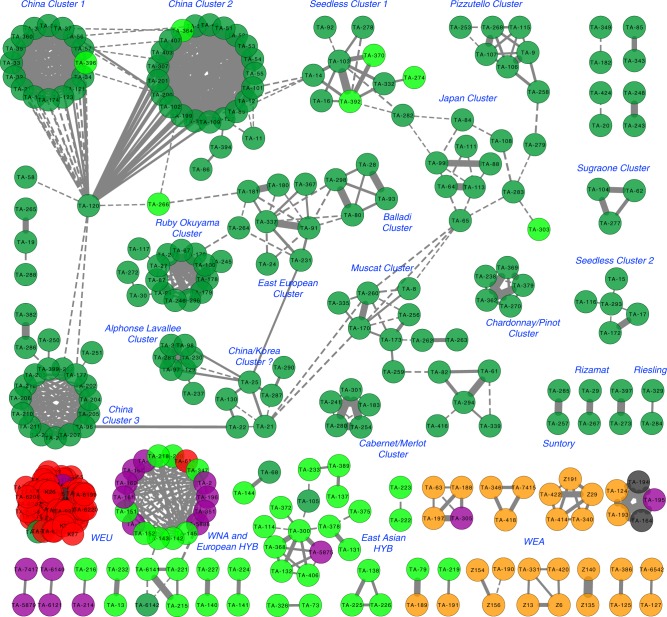
First-degree relationship network of 335 *Vitis* accessions. Dashed line represents an IBD value in between 0.420 and 0.466. Solid line represents an IBD value equal to or greater than 0.466. The thickness of the line is proportional to the calculated IBD value. Source data is provided as a Source Data file

The pedigree network via Cytoscape showed that the majority of the first-degree relationships were among accessions in the same major *Vitis* categories (Fig. 5). The wild European grapevines formed a compact stand-alone cluster. In contrast, the wild North American species were highly connected to the European interspecific hybrid cultivars, agreeing with the history that wild North American grapevines were extensively used in hybridization to counter mildews and pests in the 19th century European vineyards^34^. The wild East Asian species formed a loosely-connected cluster, which implies that wild East Asian species have yet to be fully explored for creating hybrid cultivars. The domesticated grapevine cultivars formed 16 discrete clusters. The Chinese cultivars formed three highly-connected compact clusters that were linked by the key node cultivar “Jingxiu” (Fig. 5). In comparison, the European cultivars formed loosely-connected dispersed clusters. For example, independent clusters showed first-degree relationships of “Cabernet Sauvignon/Merlot” and “Chardonnay/Pinot”, which corresponded to a previous report^10^. Another example is the formation of two separate seedless grapevine clusters, with one centered on “Thompson Seedless” and the other on “Emperor”. The network of the domesticated grapevine cultivars is an indication of a highly complicated breeding history of European domesticated grapevines that started thousands of years ago.

### Selection signals in WEU and CEU grapevines

Across the *Vitis* genome, the global nucleotide diversity (*π*) was well correlated to the global SNP and indel density (Fig. 1b). Subsequent analysis showed that the degree of polymorphism in the wild European grapevines (*π* = 3.50 × 10^−3^; S.D. = 1.84 × 10^−3^) was lower than that of the domesticated cultivars (*π* = 5.49 × 10^−3^; S.D. = 1.91 × 10^−3^). This deficit in genetic diversity in wild European grapevines versus domesticated cultivars was well noted in several independent *Vitis* studies^1,24,35^. Given that the wild European grapevines have a notable reduced *N*_*e*_ (Fig. 4), their reduced level of diversity may be the result of fragmented native habitat and increased inbreeding in the recent past^24,35^.

**Fig. 6:**
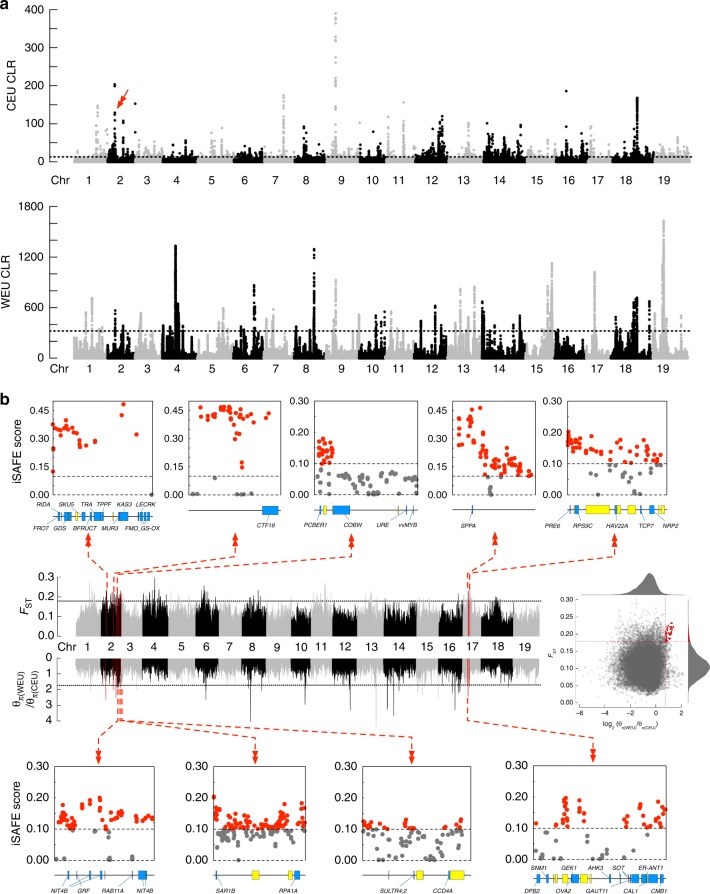
Selective sweep regions in the grapevine genome. **a** CLR scores calculated by SweeD across the genome in both wild European and domesticated grapevines. The dashed lines mark the regions at the top 0.5%. The red arrow indicates the putative sex determination region. **b** Selective sweep regions during domestication inferred from *F*_ST_ and *π* statistics and SNP hotspots in each region influenced by ongoing selection sweep. Each selective sweep region is represented by a red perpendicular line in the genome. Magnified view of each selective sweep region is represented by a horizontal line, on which annotated (blue blocks) and unknown (yellow blocks) genes are drawn. Each dot in the iSAFE plots represents the average iSAFE score for 36 SNPs (step size 18 SNPs) in the region. The dashed line shows the empirical significance cut-off. In the first iSAFE plot, this region overlaps with the putative sex determination region

Despite this drawback, we investigated potential selective signals in the genomes of wild European and domesticated grapevines by identifying the regions (about 1 kb in length) that scored top 0.5% in the CLR analysis (Fig. 6a). The 2119 selective sweep regions in the domesticated grapevine genome harbor 1016 candidate genes, whereas the 2120 selective sweep regions in the wild grapevine genome contain 348 candidate genes (Supplementary Data 3). The numbers of identified genes were larger than those found in a previous study (1016 versus 308 with 28 in common; 348 versus 88 without common genes)^6^. In particular, the previously identified flower sex determination locus on chromosome 2 (4.91–5.05 Mb^36^ or 4.88–5.04 Mb^37^) is found to contain 15 significant CLR signals in the domesticated grapevine genome. No overlapping selective sweep regions were found in the genomes of wild European and domesticated grapevines and only 18 genes were found in both lists (Supplementary Data 3). GO enrichment of the 1016 domesticated grapevine candidate genes showed significant functional representation in the GO categories of immune response, regulation of cell death, fructose metabolic process, plasma membrane, and vesicle trafficking (Supplementary Fig. 7). Interestingly, jasmonates-induced cell death was found to be associated with abiotic defense responses in grapevine^38^. Also, the vesicle trafficking of anthocyanins plays important roles in the berry skin coloration and berry ripening in grapevines^39,40^. This result suggests that domesticated grapevines may have been mainly selected for higher fructose content, various ripening times, and broader cultivation areas. In comparison, GO enrichment of 348 wild European grapevine candidate genes showed significant functional representation in the GO categories of macromolecule metabolism, aromatic compound biosynthesis, and jasmonic acid metabolic pathways, suggesting a natural selection for resistance to biotic and abiotic stresses (Supplementary Fig. 7).

Even though our wild European grapevines may not fully reflect the genetic diversity of the true progenitor wild European grapevine population, we decided to identify some potential selective signals during grapevine domestication (wild European versus domesticated cultivars) by surveying the genomic regions that showed high nucleotide diversity differences and *F*_ST_ values (both top 5%). A total of nine domestication-selective sweep regions on two chromosomes were detected, containing 73 genes (Fig. 6b, Supplementary Data 4). One of the selective sweep regions on chromosome 2 (4.83–4.97 Mb) overlaps with the previously identified flower sex determination locus^36,37^, agreeing with the aforementioned result of CLR analysis (Fig. 6b, first iSAFE plot). We then identified the SNP hotspots favored by ongoing selective sweep (SNP window size = 36, allele frequency 0.1 < *v* < 0.9) within the identified regions using iSAFE analysis (iSAFE ≥ 0.1, empirical *P* < 1 × 10^4^)^41^. The result showed that the majority of the SNP hotspots were found in the promoter side of the genes in the selective sweep regions (Fig. 6b, red dots). The functional annotation of these 73 genes revealed their putative roles in regulating cell growth and metabolism. For instance, a putative *vvMYB* gene was identified in a selective sweep region on Chr. 2 with no SNP hotspots around it. This gene has been implicated in the differences of grape skin color^42,43^, possibly through its impact on anthocyanin synthesis. A cluster of the bifunctional *NITRILASE/NITRILE HYDRATASE* (*NIT4B*) genes was also identified in a Chr. 2 selective sweep region. This gene, fairly conservative among different plant species, is involved in the detoxification of cyanide and the recovery of nitrogen from cyanogenic glycosides, which affects both plant development and plant defense^44^. Moreover, the *TRANSALDOLASE* (*GANAB*) gene and the *ACID BETA-FRUCTOFURANOSIDASE* (*BFRUCT*) gene on Chr. 2 suggest that carbohydrate metabolism is a key target of domestication-selective sweep.

### Genome-wide association analyses

Previous studies using 10–20k SNP arrays have successfully identified grapevine alleles that are associated with important phenotypes, such as seedlessness, muscat aroma, skin color, and flower sex^5,43^. Here, we performed genome-wide association analysis (GWAS), using a compressed MLM model, on the data of 24 grapevine phenotypes that were obtained over 1–3 years (Supplementary Data 5). We detected SNP signals for berry shape, number of seeds, panicle type, berry sucrose content, berry acid content, and 12 aromatic compounds, but not for berry weight, Brix score, and most of berry sugar contents (Supplementary Fig. 8–12 Supplementary Data 6, Supplementary Data 7). Among all these grapevine traits, berry shape was the only one that had been associated with SNP signals over multiple years. Specifically, five genomic loci containing nine genes (12 SNPs) were found to be associated with berry shape for the years 2016, 2017, and the pooled mean data. One exonic SNP (Chr7.20085154, C to T) causes a nonsynonymous mutation in a putative *SERINE/THREONINE-PROTEIN KINASE* (*SRK2A*) gene. Even though its physiological function in grapevine is unclear, members of the serine/threonine kinase family are regarded as the central units linking hormonal and environmental stimuli to changes in metabolism and gene expression^45^. These data suggest that it requires even larger sample sizes of grapevines to consistently discern trait-associated SNPs.

## Discussion

In this study, we reported the characterization of genome-wide SNPs from 472 *Vitis* accessions, an endeavor that covered 48 out of 60 extant *Vitis* species. The genomic variation data of this scale, though the largest ever reported for grapevine, are in no comparison to the genetic diversity of over 11,000 domesticated grapevine cultivars and wild species around the world. Preferably, this study shall act as the foundation for future more extensive collaborations on a par with the 3,000 Rice Genomes Project^46^. With this said, we were able to utilize our SNP dataset to revisit and provide information about the genetic diversity and demographic history of the domesticated grapevines.

In an ideal scenario, a true wild progenitor population is required for the delineation of grapevine domestication history and the identification of grapevine selective sweep regions in the genome. Inclusion of grapevine samples from such a population in the actual studies, however, has been proved to be difficult^6,10^. For one reason, the morphological similarity between wild European and domesticated grapevines makes the identification work prone to error^3^. Another reason is the inter-fertile nature of *Vitis* species (gene flow between two subspecies) and the possibility of feralization of the domesticated grapevines^3,35^. Moreover, the population of wild European grapevines only exist in fragmented refugia in Eurasia (e.g., Caucasus, Iberian Peninsula, Balkan Peninsula, and so on)^47^, and these subpopulations manifest substantial genetic diversity among them^10,35,47^.

In our study, the majority of the wild European samples came from the Ketsch peninsula on upper Rhine in Germany, which represented one of the largest wild European grapevine populations in Western Europe^48^. Depending on which microsatellite marker was used for analyses, the Ketsch population was genetically closer to either the Iberian population in the west or the Caucasian population in the east^48^. This result suggested that the Ketsch population might be a genetic sink for the wild European grapevines during the Ice Age^48^. Indeed, data from the phylogenetic tree and population structure analyses (Fig. 2) based on whole-genome SNPs strongly supported the majority of Ketsch population as the true wild European grapevines. Interestingly, two Ketsch accessions (KE06 and TA-6264) were found to be possible feral escapees, as they showed ambiguous phylogenetic positions and clustered with domesticated grapevine cultivars. Additionally, the previously reported wild European grapevines from Zhou et al.^6^ also clustered with our domesticated grapevines and feral escapees (Supplementary Fig. 4 and 5). Even though the Ketsch population represents a true *sylvestris* population and shares genetic diversity with the Caucasian *sylvestris* accessions^48^, we still caution against equating this Ketsch population with the true wild progenitor population.

Even though our whole-genome SNP data were not able to display the whole scheme of grapevine domestication throughout history, they were able to reveal some interesting details with regard to the demographic history of grapevines. Firstly, the divergence time between our wild European and domesticated grapevines was estimated to be around 200–400 Kya (Fig. 4), a time point that greatly predated a previous estimate (22 Kya)^6^ and the proposed grapevine domestication time (8000 ya)^9^. No matter which subpopulation of the domesticated grapevines was tested, this divergence time did not significantly change (Fig. 4). One possible explanation of the result is that the Ketsch population represents an early diverged subpopulation of wild European grapevines. The ancient progenitor population may have split from the Ketsch population (or vice versa) since 200–400 Kya, and later gave rise to all of today’s domesticated grapevines. However, the question remains whether such an ancient progenitor population survived hundreds of thousands of years till today. If it did survive, what would be the relationship between this ancient progenitor population and the wild European accessions in the Caucasus region? Secondly, the *N*_*e*_ of both wild European and domesticated grapevines experienced a continuous contraction since the Last Ice Age and a significant expansion since 20–40 Kya (Fig. 4c, d). Interestingly, the time of the *N*_*e*_ expansion corresponded to the end of the Last Ice Age^49^. A second significant *N*_*e*_ contraction occurred since 3000–4500 ya for both wild European and domesticated grapevines, and the time of the *N*_*e*_ contraction corresponded to a much-debated global drought that decimated ancient civilizations^50^. Unfortunately, there are no methods to determine if the *N*_*e*_ expanded once more in the recent 200 years. Given the magnitude and pace of *N*_*e*_ change according to our data, we would argue all grapevine populations experienced at least one severe bottleneck. Since the pattern of *N*_*e*_ change is similar between the wild European and domesticated grapevines, the bottleneck was more likely driven by global climate change, instead of human activity. This is in line with the previous conclusion that domestication-associated bottleneck (*N*_*e*_ change due to human activity) is weak^6,10^. In addition, the start of *N*_*e*_ expansion for the domesticated grapevines was earlier than that for the wild European grapevines (Fig. 4c, d). This probably reflects the early human management of the plant as a food source, lending support for a protracted domestication of grapevine starting a lot earlier than 8000 ya^6,10^. With these said, we would like to point out that more data, especially from the demographic histories of various subpopulations of wild and cultivated *Vitis* accessions, are needed to confirm global climate change as the main driver of *N*_*e*_ change. Thirdly, domesticated grapevines from the pan-Black Sea area had a distinct demographic history compared to their counterparts in other regions (Fig. 4). This is probably due to the continuous introgression of local wild European grapevines into the first domesticated grapevines as they spread across the continent. The introgression process has been widely reported in the previous studies^5,10,11,35^.

Analyses of these SNPs also shed light on a set of domestication selective sweeps that promoted grape berry edibility and aroma signatures. In addition, GWA analysis identified candidate SNP signals that are associated with an arrangement of grapevine traits. However, this approach may require data from more samples to evaluate phenotypes (e.g., berry weight, sugar content) that are highly vulnerable to environmental changes. In conclusion, this large-scale SNP resource of *Vitis* species will facilitate the breeding of new grapevine cultivars and the investigations into various aspects of grapevine biology.

## Methods

### DNA sample preparation and sequencing

347 *Vitis* accessions were obtained from the *Vitis* germplasm repository at the Institute of Botany of Chinese Academy of Sciences in Beijing, 40 accessions from the Zhengzhou Fruits Research Institute of Chinese Academy of Agricultural Sciences in Zhengzhou, and 89 accessions from the Botanical Institute of Karlsruhe Institute of Technology in Karlsruhe (Supplementary Data 1). All plants were subjected to standard management practice that included cultivation, irrigation, fertilization, pruning, and disease control. Young leaves were collected from the plants and snap frozen in liquid nitrogen. Total DNA was extracted with the DNAsecure plant kit (Tiangen, Beijing). 2 µg genomic DNA from each accession was used to construct a sequencing library following the manufacturer’s instructions using NEBNext Ultra DNA Library Prep Kit (NEB, USA). Paired-end sequencing libraries with an insert size of approximately 400 bp were sequenced on an Illumina HiSeq 4000 sequencer at Novogene-Beijing. Paired-end resequencing reads were filtered using NGSQCToolkit_v2.3.3^51^. This step removed reads containing adapter or poly-N, and low-quality reads (reads with >30% bases having Phred quality ≤25) from the raw data, yielding clean data for subsequent downstream analyses. 5 bp off the 5′ and 3′ end of a read was also trimmed.

### Variation calling and annotation

Paired-end resequencing reads were mapped to the *V. vinifera* reference genome (Ensembl Plants Release-31)^12^ with BWA (Version: 0.7.10-r789)^52^ using the default parameters. SAMtools (Version: 1.3.1)^53^ software was used to convert mapping results into the BAM format and filter the unmapped and non-unique reads. Duplicated reads were filtered with the Picard package (picard.sourceforge.net, Version: 2.1.1). After BWA alignment, the reads around indels were realigned, realignment was performed with Genome Analysis Toolkit (GATK, version 3.3-0-g37228af)^54^ in two steps. The first step used the RealignerTargetCreator package to identify regions where realignment was needed, and the second step used IndelRealigner to realign the regions found in the first step, which produced a realigned BAM file for each accession. We also downloaded the reads file for 23 grapevine accessions reported in Zhou et al.^6^ from NCBI under BioProject ID: PRJNA388292, and processed the data with same pipeline.

The variation detection followed the best practice workflow recommended by GATK^54^. In brief, the variants were called for each accession by the GATK HaplotypeCaller^54^. A joint genotyping step for comprehensive variations union was performed on the gVCF files. In the filtering step, the SNP filter expression was set as QD < 5.0 || MQ < 40.0 || FS > 60.0 || SOR > 3.0 || MQRankSum < −10.0 || ReadPosRankSum < −8.0 || QUAL < 30, and the Indel filter expression was set as QD < 2.0 || ReadPosRankSum < −10.0 || InbreedingCoeff < −0.8 || FS > 100.0 || SOR > 5.0 || QUAL < 30. Only insertions and deletions shorter than or equal to 40 bp were considered. Indels and SNPs with none bi-allelic, >40% missing calls and MAF < 0.005 were removed, which yielded the basic set. SNPs with MAF < 0.05 were further removed for phylogenetic tree structure, IBD calculation, LD decay, PCA and population structure analyses (the core set).

Copy number variations (CNVs) were detected using CNVcaller^55^. Briefly, the reference genome was segmented into overlapping 800 bp sliding windows, and the windows were indexed to form a reference database used in all samples. Then, the reads count of each window across genome from BAM file and a comparable read depth (RD) file of each individual was calculated. The normalized RD files of all samples were piled up into a two-dimensional population RD file, and the integrated CNV regions (CNVR) were detected by scanning the population RD file with aberrant RD, CNV allele frequency, and significant correlation with adjacent windows. The adjacent candidate windows showing high correlation were further merged.

SNPs and Indels annotation were performed according to the grapevine genome using the package ANNOVAR (Version: 2015-12-14)^56^. The coverage of each accession against each chromosome of grapevine genome was counted base on aligned BAM file using SAMtools (Version: 1.3.1)^53^ software. SNP density, indel density, and total genetic diversity across each chromosome were counted with 100 kb sliding window using VCFtools software (v0.1.13)^57^.

### Population genetics analysis

We used the whole-genome SNPs to construct the ML phylogenetic tree with 100 bootstrap using SNPhylo^58^ (Version: 20140701). *Parthenocissus tricuspidata* (Boston ivy) was used to provide outgroup information at corresponding positions. The tool iTOL (http://itol.embl.de) was used to color the phylogenetic tree. For each group based on the phylogenetic tree, the uncertain samples were discarded in further analyses unless specified otherwise.

SNPs in LD were filtered using PLINK (Version v1.90b3.38)^59^ with a window size of 50 SNPs (advancing 5 SNPs at a time) and an *r*^2^ threshold of 0.5. PCA was performed with the Genome-wide Complex Trait Analysis (GCTA, version: 1.25.3) software^60^, and the first three eigenvectors were plotted. Population structure was analyzed using the ADMIXTURE (Version: 1.3)^61^ program with a block-relaxation algorithm. To explore the convergence of individuals, we predefined the number of genetic clusters *K* from 2 to 8 and ran the cross-validation error (CV) procedure. Default methods and settings were used in the analyses.

LD was calculated using PopLDdecay (Version: v3.31, https://github.com/BGI-shenzhen/PopLDdecay). The pairwise *r*^2^ values within and between different chromosomes were calculated. The LD for each group was calculated using SNP pairs only from the corresponding group.

### Estimation of mutation rate in grapevines

Since previous research work^6^ used a mutation rate derived from Brassicaceae plants (*µ* = 2.5 × 10^−9^), we decided to estimate the mutation rate of grapevine. OrthoMCL^62^ were used for defining single-copy orthologous genes from five species (*V. vinifera*, *Prunus persica*, *Arabidopsis thaliana*, *Theobroma cacao*, and *Populus trichocarpa*, from Ensembl Plant Release 31). Multiple single-copy genes were aligned using Muscle^63^. The four-fold degenerate sites were extracted from each gene and concatenated into a supergene for each species to feed to MrBayes (http://mrbayes.sourceforge.net) to infer the species phylogeny using a ML approach. To estimate the divergence time of each species, the information about the fossil-calibrated divergence time between these species was collected from TimeTree (http://www.timetree.org/). The topology of the ML tree was fed to MCMCTREE in paml version 4.4^64^ for constructing a divergence time tree and calculating the divergence time (Supplementary Fig. 13). As a result, the divergence between *V. vinifera* and *P. persica* was estimated to have occurred 104 million years ago. We then identified syntenic regions between the *V. vinifera* and *P. persica* using LASTZ (http://www.bx.psu.edu/miller_lab/) with T = 2, C = 2, H = 2000, Y = 3400, L = 6000, and K = 2200. The polymorphic loci were determined according to the following standards: (1) The nucleotide from either target or query was not classified as a *N* or *n*; (2) The locus was not in an alignment gap. The sequence divergence between the *V. vinifera* and *P. persica* was estimated to be 37.2%. A mean generation time (*g*) for grapevine was set at 3 years^6,10^. The final substitution rate per nucleotide per year (*μ*) was calculated as (0.372 × 3)/(2 × 104 × 10^6^) = 5.4 × 10^−9^ mutations per year for the grapevine. This number is in line with a previous average estimate (5 × 10^−9^ to 7 × 10^−9^) for plant nuclear genes^65^.

### Demographic history reconstruction using MSMC

We employed the MSMC^32^ model to infer population size (*N*_*e*_). The input files for MSMC were generated according to MSMC Tools (https://github.com/stschiff/msmc-tools). In brief, only sites with uniquely mapped reads and sites with coverage depths between 0.5-fold and 2-fold of mean depth were used in the analyses. The remaining genomic regions were masked using the script bamCaller.py. Then all segregating sites within each group were phased using SHAPEIT (Version: v2.r837)^66^, based on a genetic map^67^. Because of the low quality of chromosome 15, an in-house genetic map of Chr. 15 was used for phasing, which was constructed using Beifeng (maternal parent). A generation time of 3 years and a mutation rate of 5.4 × 10^−9^ mutations per nucleotide per year were used to convert the scaled times and population sizes into real times and sizes. Divergence time between the WEU and CEU population was estimated using MSMC2 (https://github.com/stschiff/msmc2).

### Demographic history inference using SMC++

SMC++ (version: v1.11.1.dev0)^33^ was employed to infer population size histories and split times between the wild European grapevine and domesticated grapevine. According to the phylogenetic tree (Fig. 2a), 60 wild European grapevine accessions and 218 domesticated grapevine accessions (tetraploid excluded) were used for the SMC++ analysis. For the split analysis, 14 cultivars from the pan-Black Sea, Western European, and East Asian regions were used together with 14 WEU samples. We performed the analysis by masking all selective sweep regions (see below). A generation time of 3 years and a mutation rate of 5.4 × 10^−9^ mutations per nucleotide per year were used to convert the scaled times and population sizes into real times and sizes.

### Pedigree construction

We calculated IBD for all pairwise comparisons among the 476 *Vitis* accessions using PLINK^59^ according to a method published elsewhere^10^. Pairs of accessions are considered to be genetically identical, if they had an IBD > 95%. We used the distribution pattern of all pairwise IBD values (Supplementary Fig. 6a) to determine the cut-off value for first-degree relatives (IBD value ≥ 0.42). We also used the lowest pairwise IBD value (≥0.466) from 43 confirmed parent–offspring relationships mentioned in a previous study as a more stringent empirical cut-off^10^. The network images base on IBD were generated using Cytoscape (Version 3.6.0, http://www.cytoscape.org/).

### Genome scanning for selective sweep signals

SweeD (Version 3.3.1)^68^ was used to detect selective sweeps based on the CLR test to detect signatures of artificial selection and natural selection in WEU and CEU accessions, respectively. We also performed a genetic differentiation (*F*_ST_) and polymorphism levels (*θπ*, pairwise nucleotide variation as a measure of variability) based cross approach to investigate the selection signals across the whole genome. A 100 kb sliding window with 10 kb step approach was applied to quantify *F*_ST_ and *θπ* by using VCFtools software (v0.1.13)^57^. The candidates that meet both top 5% of the two values were selected as selective signals.

The specific mutation favored by selection in selective sweeps (identified by the *θπ* and *F*_ST_ cross approach) was captured with phased genotypes using iSAFE (v1.0)^41^ with parameter --window 36 --step 18 --MaxRank 2 --MaxFreq 0.9.

### Grapevine traits collection

The panicle type was collected by direct observation and judgement. About 50 ripe berries were collected from each available *Vitis* accession. After berry weight and berry shape were recorded, the samples were crushed in a hand juicer where the number of seeds was counted. The must was collected in a 50 ml centrifuge tube and centrifuged at 5000*g* for 6 min. The supernatant was snap frozen in liquid nitrogen and stored at 40 °C for later analysis of sugars and acid contents. 1 ml of each sample was passed through a Supelclean^TM^ ENVI LC-18 SPE cartridge (Sigma-Aldrich, St. Louis, USA), and then diluted to 5 ml with distilled water. The diluted sample was passed through a 0.22 μm membrane filter. The sugar and acid contents were analyzed using a Dionex P680 HPLC system. The sugar contents were detected using a Shodex RI-101 refractive index detector with reference cell maintained at 40 °C. The Sugar-Pak I column (300 mm × 6.5 mm I.D., 10 μm particle size, Waters, USA) with a Sugar-Pak I Guard-PakInsert (10 μm particle size, Waters, USA) was used. The column was maintained at 90 °C with a Dionex TCC-100 thermostat column compartment. Samples were eluted with double-distilled water. The flow rate was 0.6 ml/min. The Chromeleon chromatography data system was used to integrate peak areas according to external standard solution (Sigma-Aldrich, St. Louis, USA). Malic and tartaric acid contents were detected using a Dionex PDA-100 detector. The Dikma PLATISIL ODS column (250 mm × 4.6 mm I.D., 5 μm particle size, Dikma, China) with a DikmaSpursil C18 Guard Cartridge (3 μm, 10 mm × 2.1 mm, Dikma, China) was used. The column was maintained at 40 °C. Samples were eluted with 0.02 mol/L KH_2_PO_4_ solution at pH 2.4. The flow rate was 0.8 ml/min. Eluted compounds were detected by UV absorbance at 210 nm. Acid concentration was determined according to external standard solution calibrations.

A new batch of grape berry samples were snap frozen in liquid nitrogen and stored at −40 °C. The frozen berries were crushed with a mortar and a pestle to remove seeds. The flesh and skin tissues were ground in an IKA A11 mill (IKA Works Inc., Germany) while frozen. A puree was prepared by pulverizing 50 g pitted frozen berries with 5 g CaCl_2_ to decrease the rate of enzymatic reactions. A 1 cm SPME fiber coated with 50/30 μm divinylbenzene/carboxen/polydimethylsiloxane (Supelco Inc., Bellefonte, PA) was used to conduct the headspace solid-phase microextraction. Five grams of the puree was placed in a 20 ml capped vial with 10 µl of 32.84 mg/L 3-octanol/ethanol solution, which was used as an internal standard for quantification. The samples were stirred at 40 °C and after 20 min of equilibration between the solution and the headspace, the fiber was exposed to the headspace of the capped vial for a period of 30 min. The fiber was then withdrawn and introduced into the injection port of the GC for desorption at 250 °C for 4 min in the splitless mode.

### GC-MS analysis

Qualitative analysis of the volatile compounds was performed according to a modified method described elsewhere^69^. In brief, the analysis was performed using an Agilent 7890 gas chromatograph equipped with a DB-17MS capillary column (30 m × 0.25 mm × 0.25 µm; J&W, Folsom, CA), coupled to an Agilent 5975C quadrupole mass spectrometer (Agilent, Santa Clara, CA). The oven program was as follows: 40 °C for 5 min, 40–70 °C with gradual increase of 2 °C/min, 70 °C for 2 min, 70–120 °C with gradual increase of 3 °C/min, 120–150 °C with gradual increase of 5 °C/min, 150–220 °C at 10 °C/min, and then 220 °C for 2 min. The injector temperature was maintained at 250 °C, and the transfer line temperature was 280 °C. The ion source temperature was 230 °C. The electronic impact (EI) was 70 eV, scanned in the range of *m*/*z* 30–300 at a rate of 2.88 scans/s. Helium was employed as a carrier gas, and was introduced at a flow rate of 1 ml/min. A tentative identification of volatile compounds present was achieved by comparing the observed mass spectra with the data system library (NIST2008) and published spectra (Mass Spectrometry Data Center, 1974), supported by retention index data, which were then compared against available literature listing known retention indices (NIST Chemistry WebBook, 2005). All compounds were quantified as 3-octanol equivalents.

### Genome-wide association analysis

To minimize false positives and increase statistical power, population structure and cryptic relationships were considered. A compressed mixed linear model program, GAPIT (Version: 2016.03.01)^70^ was used for the association analysis. The chromosome SNPs were further filtered by a maximum missing rate greater than 30% and MAF < 0.05, and the cluster SNPs (3 SNPs exit within 10 bp) were also removed. The first three PCA values (eigenvectors), which were derived from whole-genome SNPs, were used as fixed effects in the mixed model to correct for stratification^71^.

We defined the whole-genome significance cutoff with the adjusted Bonferroni test threshold, which was set as *P* < 0.05/total SNPs. For aroma traits, there are 8,734,701 SNPs from 185 accessions, therefore log_10_ (*P*) = −8.24. For SUC, TAR, MAL, TA, and Panicle type traits, there are 9,068,232 SNPs from 222 accessions, therefore log_10_ (*P*) = −8.26. For Color, Weight, Shape, Brix, Seed traits, there are 9,191,395 SNPs from 334 accessions, therefore log_10_ (*P*) = −8.26.

### Reporting summary

Further information on experimental design is available in the Nature Research Reporting Summary linked to this article.

## Supplementary information

Supplementary Information

Reporting Summary

Description of Additional Supplementary Files

Supplementary Data 1

Supplementary Data 2

Supplementary Data 3

Supplementary Data 4

Supplementary Data 5

Supplementary Data 6

Supplementary Data 7

Source Data

## Data Availability

The WGRS data set generated and analyzed in the current study is available from NCBI under the BioProject accession PRJNA393611. Data supporting the findings of this work are available within the paper and its Supplementary Information files. The source data of Fig. 5 and Supplementary Figs. 2 and 6 are provided as a Source Data file. All data are available from the corresponding author upon reasonable request. A reporting summary for this article is available as a Supplementary Information file.

## References

[1] Emanuelli F (2013). Genetic diversity and population structure assessed by SSR and SNP markers in a large germplasm collection of grape. BMC Plant Biol..

[2] Janick, J. & Paull, R. E. *The Encyclopedia of Fruit and Nuts* (CABI Publishing, Oxford, United Kingdom, 2008).

[3] This P, Lacombe T, Thomas MR (2006). Historical origins and genetic diversity of wine grapes. Trends Genet..

[4] Myles S (2010). Rapid genomic characterization of the genus *vitis*. PLoS One.

[5] Laucou V (2018). Extended diversity analysis of cultivated grapevine *Vitis vinifera* with 10K genome-wide SNPs. PLoS One.

[6] Zhou Y, Massonnet M, Sanjak JS, Cantu D, Gaut BS (2017). Evolutionary genomics of grape (*Vitis vinifera* ssp. vinifera) domestication. Proc. Natl. Acad. Sci. U.S.A..

[7] Tabidze V (2017). Whole genome comparative analysis of four Georgian grape cultivars. Mol. Genet. Genomics.

[8] Mercenaro L, Nieddu G, Porceddu A, Pezzotti M, Camiolo S (2017). Sequence polymorphisms and structural variations among four grapevine (*Vitis vinifera* L.) cultivars representing Sardinian agriculture. Front. Plant Sci..

[9] McGovern P (2017). Early Neolithic wine of Georgia in the South Caucasus. Proc. Natl. Acad. Sci. U.S.A..

[10] Myles S (2011). Genetic structure and domestication history of the grape. Proc. Natl. Acad. Sci. U.S.A..

[11] Arroyo-Garcia R (2006). Multiple origins of cultivated grapevine (*Vitis vinifera* L. ssp. sativa) based on chloroplast DNA polymorphisms. Mol. Ecol..

[12] Jaillon O (2007). The grapevine genome sequence suggests ancestral hexaploidization in major angiosperm phyla. Nature.

[13] Duan N (2017). Genome re-sequencing reveals the history of apple and supports a two-stage model for fruit enlargement. Nat. Commun..

[14] Wang X (2017). Genomic analyses of primitive, wild and cultivated citrus provide insights into asexual reproduction. Nat. Genet..

[15] Varshney RK (2017). Whole-genome resequencing of 292 pigeonpea accessions identifies genomic regions associated with domestication and agronomic traits. Nat. Genet..

[16] Aflitos S (2014). Exploring genetic variation in the tomato (Solanum section Lycopersicon) clade by whole-genome sequencing. Plant J..

[17] Zhou Z, Jiang Y (2015). Resequencing 302 wild and cultivated accessions identifies genes related to domestication and improvement in soybean. Nat. Biotechnol..

[18] 3,000 Rice Genomes Project. (2014). The 3,000 rice genomes project. Gigascience.

[19] Jun Wen (2018). Chloroplast phylogenomics of the New World grape species (*Vitis*, Vitaceae). J. Syst. Evol..

[20] Klein LL (2018). High-throughput sequencing data clarify evolutionary relationships among North American *Vitis* species and improve identification in USDA *Vitis* germplasm collections. Am. J. Bot..

[21] Wan Y (2013). A phylogenetic analysis of the grape genus (*Vitis* L.) reveals broad reticulation and concurrent diversification during neogene and quaternary climate change. BMC Evol. Biol..

[22] Trondle D (2010). Molecular phylogeny of the genus *Vitis* (Vitaceae) based on plastid markers. Am. J. Bot..

[23] Remington DL (2001). Structure of linkage disequilibrium and phenotypic associations in the maize genome. Proc. Natl. Acad. Sci. U.S.A..

[24] Marrano A, Micheletti D, Lorenzi S, Neale D, Grando MS (2018). Genomic signatures of different adaptations to environmental stimuli between wild and cultivated *Vitis vinifera* L. Hortic. Res..

[25] Nicolas SD (2016). Genetic diversity, linkage disequilibrium and power of a large grapevine (*Vitis vinifera* L) diversity panel newly designed for association studies. BMC Plant Biol..

[26] Huang X (2012). A map of rice genome variation reveals the origin of cultivated rice. Nature.

[27] Hufford MB (2012). Comparative population genomics of maize domestication and improvement. Nat. Genet..

[28] Beissinger TM (2016). Recent demography drives changes in linked selection across the maize genome. Nat. Plants.

[29] Wang L (2017). The interplay of demography and selection during maize domestication and expansion. Genome Biol..

[30] Meyer RS (2016). Domestication history and geographical adaptation inferred from a SNP map of African rice. Nat. Genet..

[31] Cubry P (2018). The rise and fall of African rice cultivation revealed by analysis of 246 new genomes. Curr. Biol..

[32] Schiffels S, Durbin R (2014). Inferring human population size and separation history from multiple genome sequences. Nat. Genet..

[33] Terhorst J, Kamm JA, Song YS (2017). Robust and scalable inference of population history from hundreds of unphased whole genomes. Nat. Genet..

[34] De Andres MT (2012). Genetic diversity of wild grapevine populations in Spain and their genetic relationships with cultivated grapevines. Mol. Ecol..

[35] Riaz S (2018). Genetic diversity analysis of cultivated and wild grapevine (*Vitis vinifera* L.) accessions around the Mediterranean basin and Central Asia. BMC Plant Biol..

[36] Fechter I (2012). Candidate genes within a 143 kb region of the flower sex locus in *Vitis*. Mol. Genet. Genomics.

[37] Picq S (2014). A small XY chromosomal region explains sex determination in wild dioecious *V. vinifera* and the reversal to hermaphroditism in domesticated grapevines. BMC Plant Biol..

[38] Repka V, Čarná M, Pavlovkin J (2013). Methyl jasmonate-induced cell death in grapevine requires both lipoxygenase activity and functional octadecanoid biosynthetic pathway. Biologia.

[39] Gomez C (2011). In vivo grapevine anthocyanin transport involves vesicle-mediated trafficking and the contribution of anthoMATE transporters and GST. Plant J..

[40] Kuhn N (2014). Berry ripening: recently heard through the grapevine. J. Exp. Bot..

[41] Akbari A (2018). Identifying the favored mutation in a positive selective sweep. Nat. Methods.

[42] Yakushiji H (2006). A skin color mutation of grapevine, from black-skinned Pinot Noir to white-skinned Pinot Blanc, is caused by deletion of the functional VvmybA1 allele. Biosci. Biotechnol. Biochem..

[43] Migicovsky Z (2017). Patterns of genomic and phenomic diversity in wine and table grapes. Hortic. Res..

[44] Jenrich R (2007). Evolution of heteromeric nitrilase complexes in Poaceae with new functions in nitrile metabolism. Proc. Natl. Acad. Sci. U.S.A..

[45] Hardie DG (1999). Plant protein serine/threonine kinases: classification and functions. Annu. Rev. Plant Physiol. Plant Mol. Biol..

[46] Wang W (2018). Genomic variation in 3,010 diverse accessions of Asian cultivated rice. Nature.

[47] Fabrizio G, Fabrizio DM, Giovanni Z, Francesco S, Massimo L (2008). Historical isolation and quaternary range expansion of divergent lineages in wild grapevine. Biol. J. Linn. Soc..

[48] Nick, P. Schützen und nützen—von der erhaltung zur anwendung. Fallbeispiel europäische wildrebe. In *Handbuch Genbank WEL: HOPPEA Denkschriften der Regensburgischen Botanischen Gesellschaft Sonderband* (Verlag der gesellschaft, Regensburg, 2014).

[49] Hewitt G (2000). The genetic legacy of the Quaternary ice ages. Nature.

[50] deMenocal PB (2001). Cultural responses to climate change during the late Holocene. Science.

[51] Patel RK, Jain M (2012). NGS QC Toolkit: a toolkit for quality control of next generation sequencing data. PLoS One.

[52] Li H, Durbin R (2009). Fast and accurate short read alignment with Burrows–Wheeler transform. Bioinformatics.

[53] Li H (2009). The sequence alignment/map format and SAMtools. Bioinformatics.

[54] McKenna A (2010). The Genome Analysis Toolkit: a MapReduce framework for analyzing next-generation DNA sequencing data. Genome Res..

[55] Wang X (2017). CNVcaller: highly efficient and widely applicable software for detecting copy number variations in large populations. Gigascience.

[56] Wang K, Li M, Hakonarson H (2010). ANNOVAR: functional annotation of genetic variants from high-throughput sequencing data. Nucleic Acids Res..

[57] Danecek P (2011). The variant call format and VCFtools. Bioinformatics.

[58] Lee TH, Guo H, Wang X, Kim C, Paterson AH (2014). SNPhylo: a pipeline to construct a phylogenetic tree from huge SNP data. BMC Genomics.

[59] Purcell S (2007). PLINK: a tool set for whole-genome association and population-based linkage analyses. Am. J. Hum. Genet..

[60] Yang J, Lee SH, Goddard ME, Visscher PM (2011). GCTA: a tool for genome-wide complex trait analysis. Am. J. Hum. Genet..

[61] Alexander DH, Novembre J, Lange K (2009). Fast model-based estimation of ancestry in unrelated individuals. Genome Res..

[62] Li L, Stoeckert CJ, Roos DS (2003). OrthoMCL: identification of ortholog groups for eukaryotic genomes. Genome Res..

[63] Edgar RC (2004). MUSCLE: multiple sequence alignment with high accuracy and high throughput. Nucleic Acids Res..

[64] Yang Z (2007). PAML 4: phylogenetic analysis by maximum likelihood. Mol. Biol. Evol..

[65] Wolfe KH, Sharp PM, Li WH (1989). Rates of synonymous substitution in plant nuclear genes. J. Mol. Evol..

[66] Delaneau O, Marchini J, Zagury JF (2011). A linear complexity phasing method for thousands of genomes. Nat. Methods.

[67] Yang S (2016). Next generation mapping of enological traits in an F2 interspecific grapevine hybrid family. PLoS One.

[68] Pavlidis P, Zivkovic D, Stamatakis A, Alachiotis N (2013). SweeD: likelihood-based detection of selective sweeps in thousands of genomes. Mol. Biol. Evol..

[69] Yang C (2009). Volatiles of grape berries evaluated at the germplasm level by headspace-SPME with GC–MS. Food Chem..

[70] Lipka AE (2012). GAPIT: genome association and prediction integrated tool. Bioinformatics.

[71] Price AL (2006). Principal components analysis corrects for stratification in genome-wide association studies. Nat. Genet..

