# Author Correction: Whole-genome resequencing of 472 *Vitis* accessions for grapevine diversity and demographic history analyses

**DOI:** 10.1038/s41467-020-16254-0

**Published:** 2020-05-06

**Authors:** Zhenchang Liang, Shengchang Duan, Jun Sheng, Shusheng Zhu, Xuemei Ni, Jianhui Shao, Chonghuai Liu, Peter Nick, Fei Du, Peige Fan, Ruzhi Mao, Yifan Zhu, Weiping Deng, Min Yang, Huichuan Huang, Yixiang Liu, Yiqing Ding, Xianju Liu, Jianfu Jiang, Youyong Zhu, Shaohua Li, Xiahong He, Wei Chen, Yang Dong

**Affiliations:** 1 Beijing Key Laboratory of Grape Sciences and Enology, Laboratory of Plant Resources, Institute of Botany, Chinese Academy of Sciences, Beijing, 100093 China; 2 Sino-Africa Joint Research Center, Chinese Academy of Sciences, Wuhan, 430074 China; 3 State Key Laboratory for Conservation and Utilization of Bio-Resources in Yunnan, Yunnan Agricultural University, Kunming, 650201 China; 4 Nowbio Biotechnology Company, Kunming, 650201 China; 5 Yunnan Research Institute for Local Plateau Agriculture and Industry, Kunming, 650201 China; 6 Key Laboratory for Agro-biodiversity and Pest Control of Ministry of Education, Yunnan Agricultural University, Kunming, 650201 China; 7 BGI, BGI-Shenzhen, Shenzhen, 518120 China; 8 BGI Institute of Applied Agriculture, BGI-Shenzhen, Shenzhen, 518120 China; 9 Zhengzhou Fruit Research Institute, Chinese Academy of Agricultural Sciences, Zhengzhou, 450009 China; 10 Botanical Institute, Karlsruhe Institute of Technology, Karlsruhe, 76128 Germany; 11 University of Chinese Academy of Sciences, Beijing, 100049 China

**Keywords:** Agricultural genetics, Genetic variation, DNA sequencing, Plant evolution

Correction to: *Nature Communications* 10.1038/s41467-019-09135-8, published online 13 March 2019.

In the original version of this Article, in the last paragraph of the first section of the Results, the ratios of SNPs located in the intergenic regions, SNPs located in the coding sequences, and indels located in the coding regions are incorrectly written as ‘71.2%’, ‘4.6%’, and ‘1.4%’, respectively. These ratios should read as ‘73.7%’, ‘4.0%’, and ‘1.3%’, respectively. In addition, the estimated ratio of indels in the coding regions that could cause frameshift mutations is incorrectly written as ‘66.7%’. It should read as ‘66.9%’. Furthermore, authors’ given names and surnames of Reference 47 are switched. The authors’ names should read ‘Grassi F., De Mattia F., Zecca G., Sala F., & Labra M.’. These have been corrected in both the PDF and HTML versions of the Article.

