# Supplementary material for: Whole-genome resequencing of 472 Vitis accessions for grapevine diversity and demographic history analyses
Source: Nat Commun. 2019 Mar 13;10:1190. doi: 10.1038/s41467-019-09135-8 (PMC6416300; doi:10.1038/s41467-019-09135-8)
Supplement: Supplementary file 1 — Supplementary Information [file 41467_2019_9135_MOESM1_ESM.pdf]

Whole-genome resequencing of 472 *Vitis* accessions for grapevine diversity and demographic history analyses

Liang *et al.*

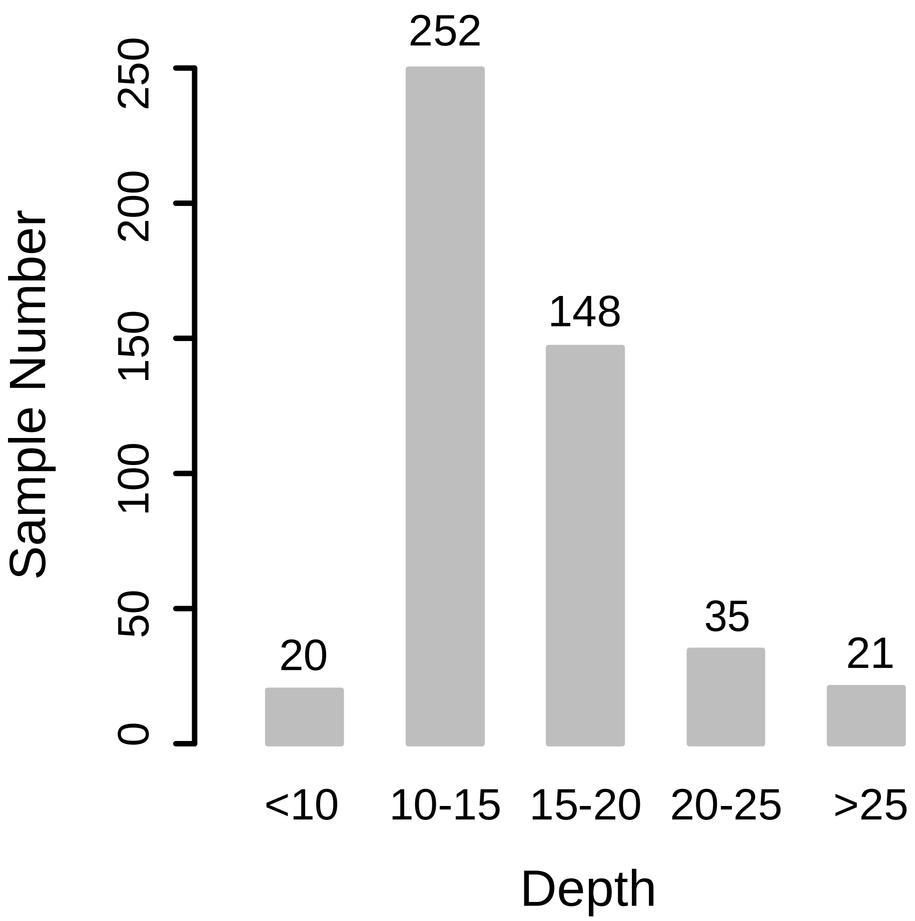

Supplementary Figure 1 Sequencing depth of grapevine accessions.

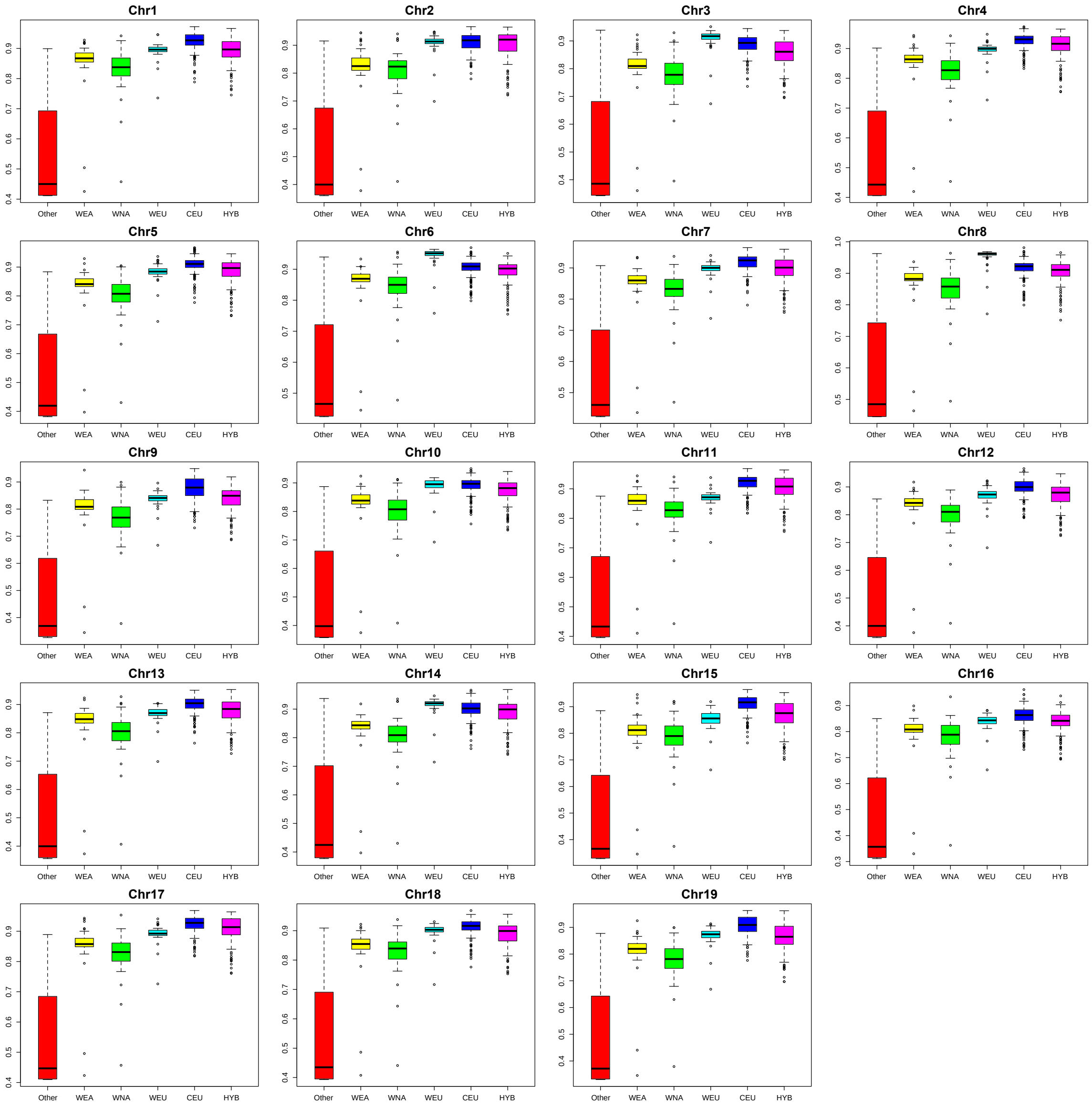

Supplementary Figure 2 Whisker-Box plots of the coverage of accessions for every chromosome of the grapevine genome. WNA, WEA, WEU, CEU, and HYB represent wild North American *Vitis* species, wild East Asian *Vitis* species, wild European grapevine, domesticated grapevine cultivars, and interspecific-hybrid grapevine cultivars, respectively. Other includes o1 (*Parthenocissus tricuspidata*), TA-164 (*Ampelopsis glandulosa var. brevipedunculata*), TA-194 (*Cayratia japonica*) and TA-5887 (*Muscadinia rotundifolia*) accession. Source data is provided as a Source Data file.

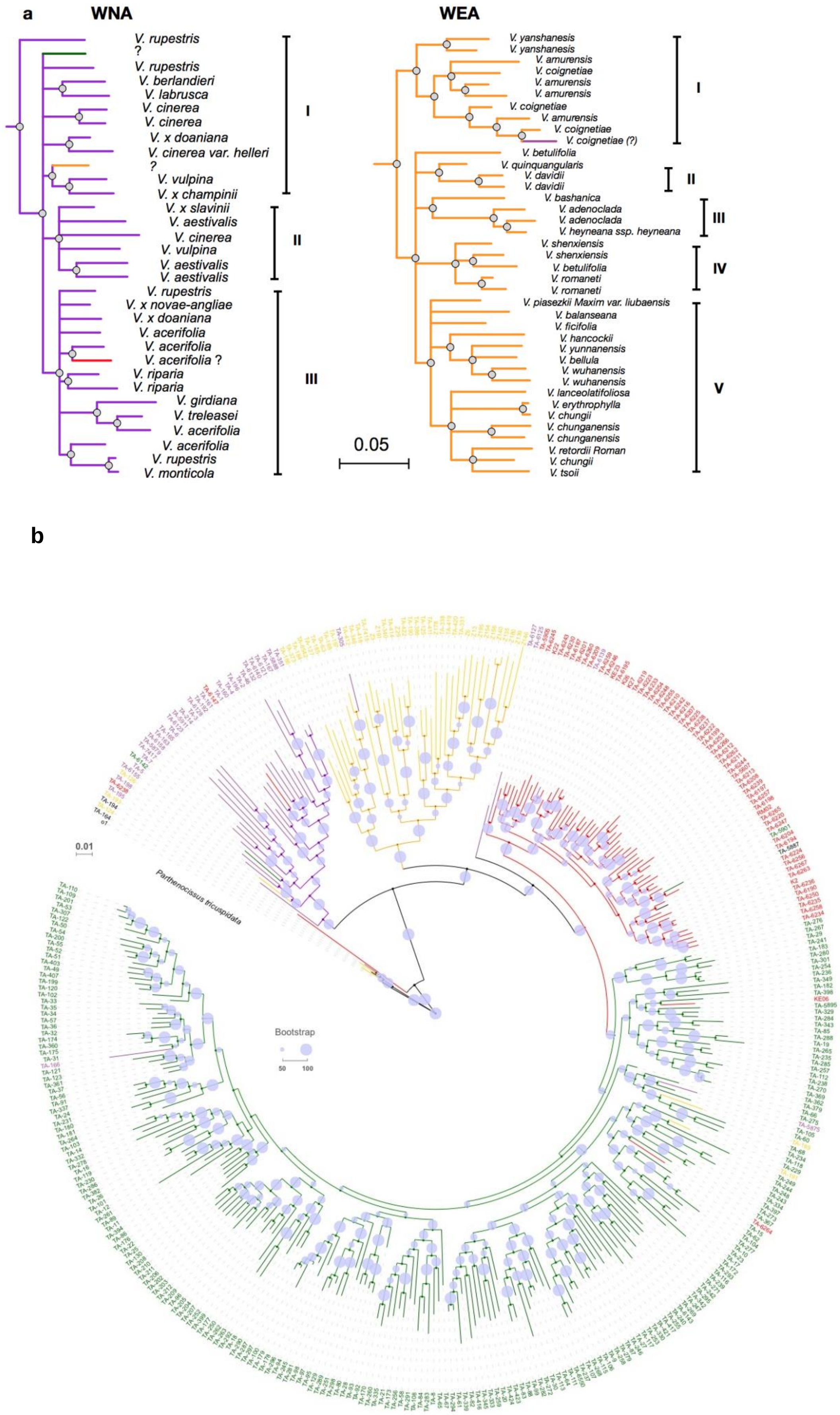
a
WNA
V. rupestris
?
V. rupestris
V. berlandieri
V. labrusca
V. cinerea
V. cinerea
V. x doaniana
V. cinerea var. helleri
?
V. vulpina
V. x champinii
V. x slavinii
V. aestivalis
V. cinerea
V. vulpina
V. aestivalis
V. aestivalis
V. rupestris
V. x novae-angliae
V. x doaniana
V. acerifolia
V. acerifolia
V. acerifolia ?
V. riparia
V. riparia
V. girdiana
V. treleasei
V. acerifolia
V. acerifolia
V. rupestris
V. monticola
I
II
III
WEA
V. yanshanesis
V. yanshanesis
V. amurensis
V. coignetiae
V. amurensis
V. amurensis
V. coignetiae
V. amurensis
V. coignetiae
V. coignetiae (?)
V. betulifolia
V. quinquangularis
V. davidii
V. davidii
V. bashanica
V. adenoclada
V. adenoclada
V. heyneana ssp. heyneana
V. shenxiensis
V. shenxiensis
V. betulifolia
V. romaneti
V. romaneti
V. piasezkii Maxim var. liubaensis
V. balanseana
V. ficifolia
V. hancockii
V. yunnanensis
V. bellula
V. wuhanensis
V. wuhanensis
V. lanceolatifoliosa
V. erythrophylla
V. chungii
V. chunganensis
V. chunganensis
V. retordii Roman
V. chungii
V. tsoii
I
II
III
IV
V
0.05
b
0.01
Parthenocissus tricuspidata
Bootstrap
50
100

Supplementary Figure 3 Maximum Likelihood phylogenetic tree of wild North American *Vitis* species (WNA), wild East Asian *Vitis* species (WEA), wild European grapevines (WEU), and cultivated grapevines (CEU). *Parthenocissus tricuspidata* (Bosten ivy) as an outgroup. Red, dark green, purple and yellow represent wild European grapevine, domesticated grapevine cultivars, wild North American *Vitis* species and wild East Asian *Vitis* species, and respectively. (a) Zoomed in view of WNA and WEA species. Grey dots represent nodes with a bootstrap value greater than 75. (b) Overview of the phylogenetic tree with bootstrap values indicated by blue circles.

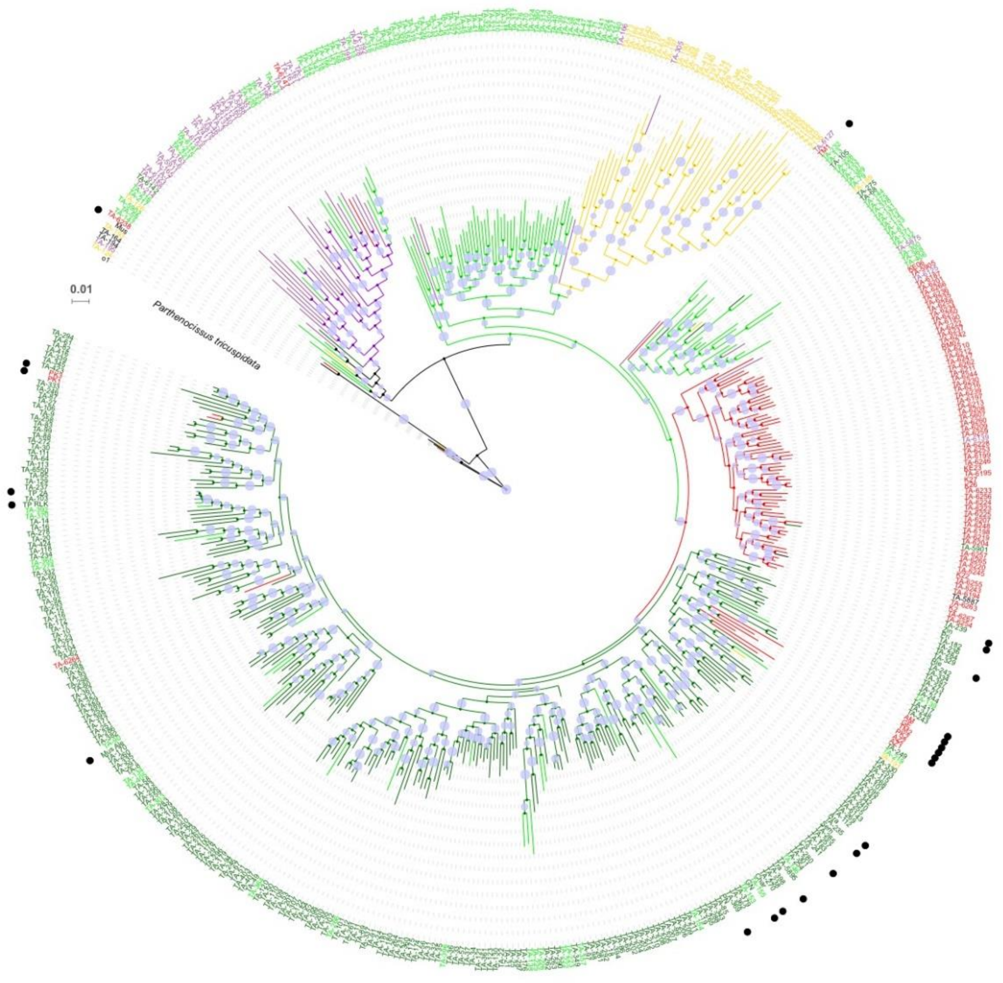

Supplementary Figure 4 Maximum Likelihood phylogenetic tree of all accessions inferred from whole-genome SNPs, with *Parthenocissus tricuspidata* (Bosten ivy) as outgroup. Red, dark green, light green, purple and yellow represent wild European grapevine, domesticated grapevine cultivars, interspecific-hybrid grapevine cultivars, wild North American *Vitis* species and wild East Asian *Vitis* species, and respectively. Bootstrap values are indicated by blue circles. Accessions marked by black dots are from Zhou *et al.* (PNAS, 2017).

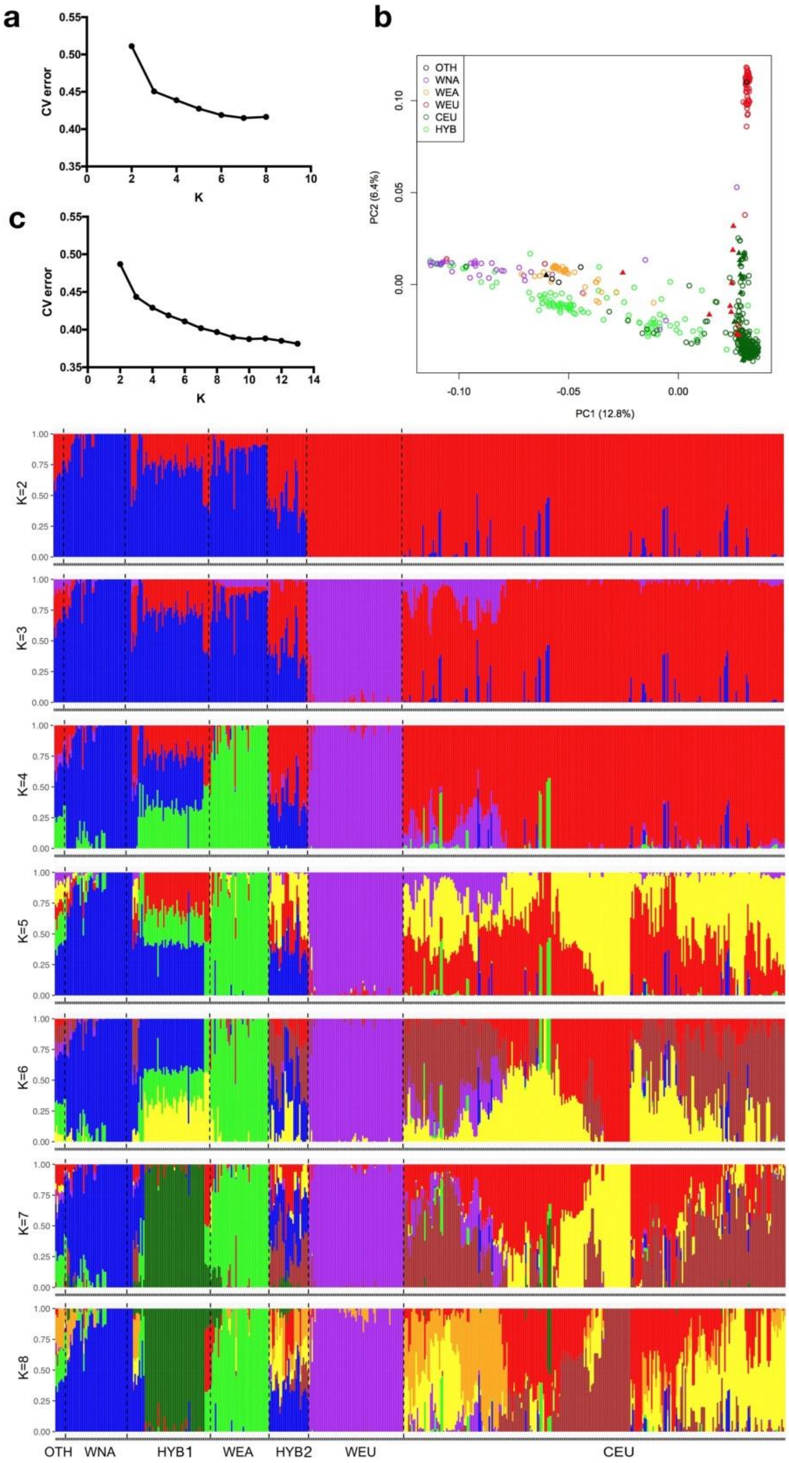
a
CV error
K
b
OTH
WNA
WEA
WEU
CEU
HYB
PC2 (6.4%)
PC1 (12.8%)
c
CV error
K
K=2
K=3
K=4
K=5
K=6
K=7
K=8
OTH
WNA
HYB1
WEA
HYB2
WEU
CEU

Supplementary Figure 5 Population structure analyses of *Vitis* accessions. (a). Cross-validation error estimate plot for the population structure, relating to Fig. 2c. When K is greater than 6, the variation of CV error is slight, and the change of proportion contributed by ancestral populations mainly exists inside of CEU, so K = 6 is considered a sensible modeling choice. (b). PCA analysis of all *Vitis* accessions in this study (indicated by circles) and *Vitis* accessions from Zhou et al (PNAS, 2017; indicated by triangles) (c). Population admixture of all *Vitis* accessions. Each color represents one ancestral population. Each accession is represented by a vertical bar, and the length of each colored segment in each vertical bar represents the proportion contributed by ancestral populations. The order of each accession was the same as that of the phylogenetic tree of whole-genome SNPs in Supplementary Fig. 4. Cross-validation error estimate plot shows that K = 8 is the optimal value. WNA, WEA, WEU, CEU, and HYB represent wild North American *Vitis* species, wild East Asian *Vitis* species, wild European grapevine, domesticated grapevine cultivars, and interspecific-hybrid grapevine cultivars, respectively.

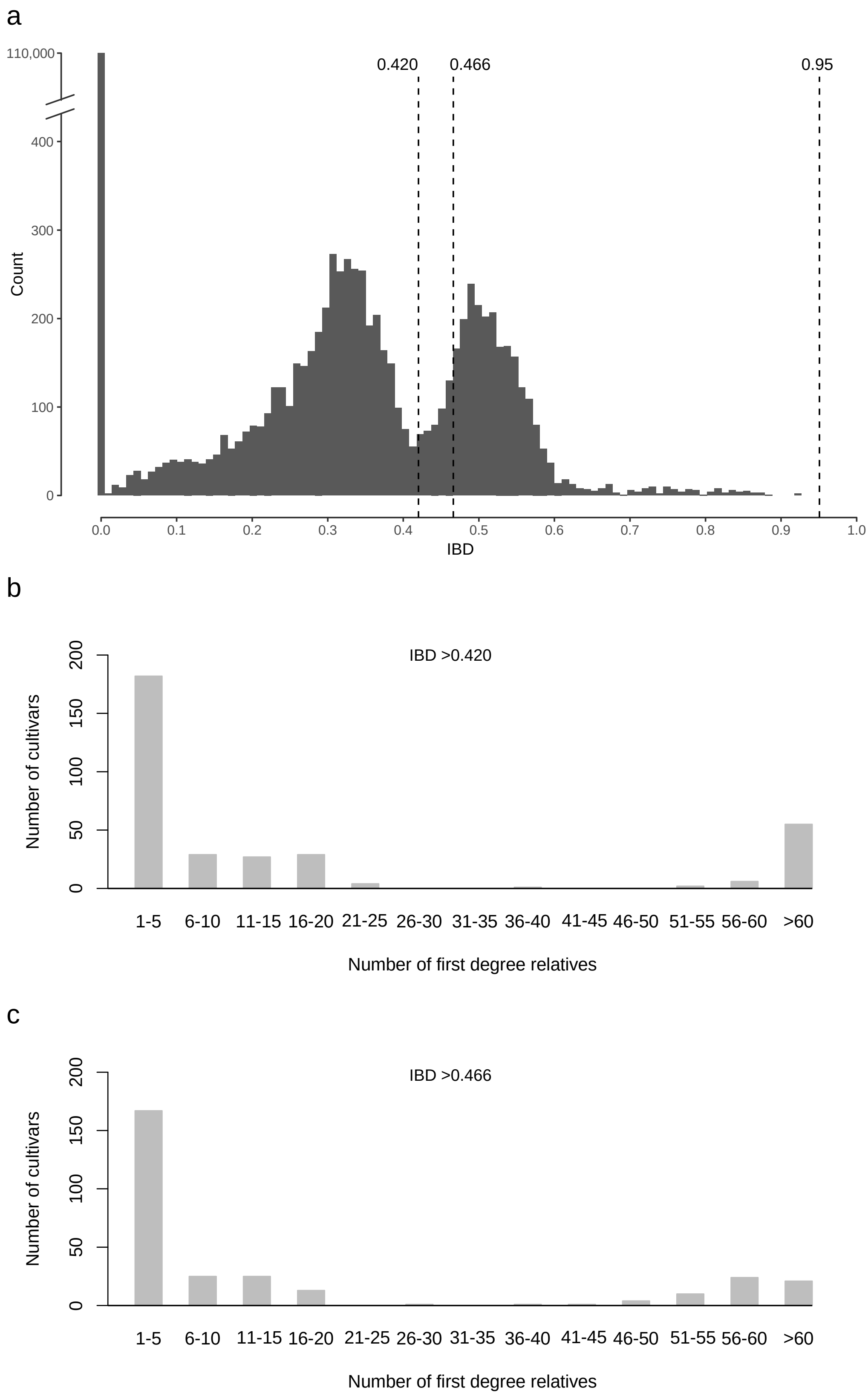
a
110,000
400
300
200
100
0
Count
0.420
0.466
0.95
0.0
0.1
0.2
0.3
0.4
0.5
0.6
0.7
0.8
0.9
1.0
IBD
b
IBD >0.420
Number of cultivars
0
50
100
150
200
1-5
6-10
11-15
16-20
21-25
26-30
31-35
36-40
41-45
46-50
51-55
56-60
>60
Number of first degree relatives
c
IBD >0.466
Number of cultivars
0
50
100
150
200
1-5
6-10
11-15
16-20
21-25
26-30
31-35
36-40
41-45
46-50
51-55
56-60
>60
Number of first degree relatives

Supplementary Figure 6 Summary statistics of identity-by-descent (IBD) values calculated from all *Vitis* accessions. (a) Histogram of IBD values from pairwise comparisons among grapevine accessions. The IBD values for a large majority of pairs of accessions were close to zero. Dashed lines represent an IBD value of 0.420 (lowest value that separates two modes), 0.466 (empirical value from Myles et al.), and 0.950 (empirical cut-off value for excluding clonality, from Myles et al.). No cultivars with ≥ 0.950 (effectively genetically identical) were found in this study. (b) Number of first-degree relationships with IBD cut-off of 0.420. 335 *Vitis* accessions (71.0%) were related to at least one other accession by a first-degree relationship. (c). Number of first-degree relationships with IBD cut-off of 0.466. 292 *Vitis* accessions (61.9%) retained a first-degree relationship with at least one other accession. Source data is provided as a Source Data file.

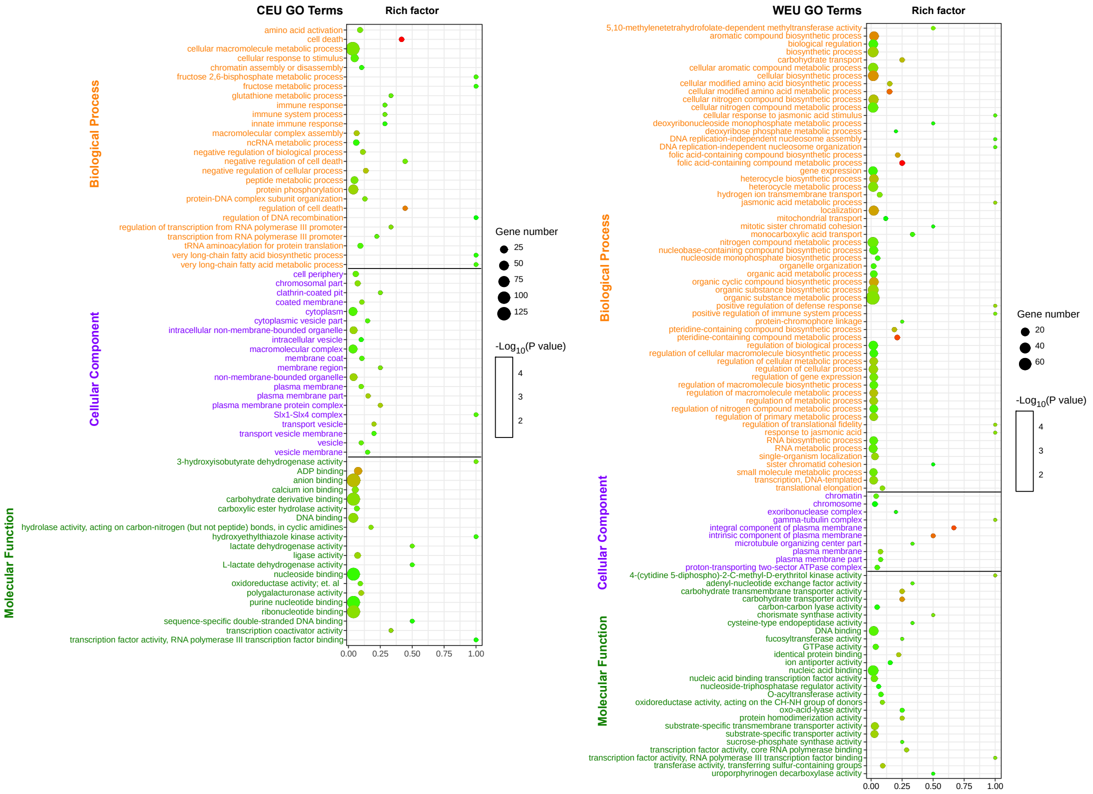

Supplementary Figure 7 GO enrichment of the candidate selective sweep genes from the CLR analysis in the domesticated grapevines (CEU, n = 1,016) and wild European grapevines (WEU, n = 348). The size of the bubble represents the number of genes in the corresponding GO category. The color of the bubble shows the corresponding *P*-value. Rich factor shows the percentage of enriched genes out of the total number in the GO category.

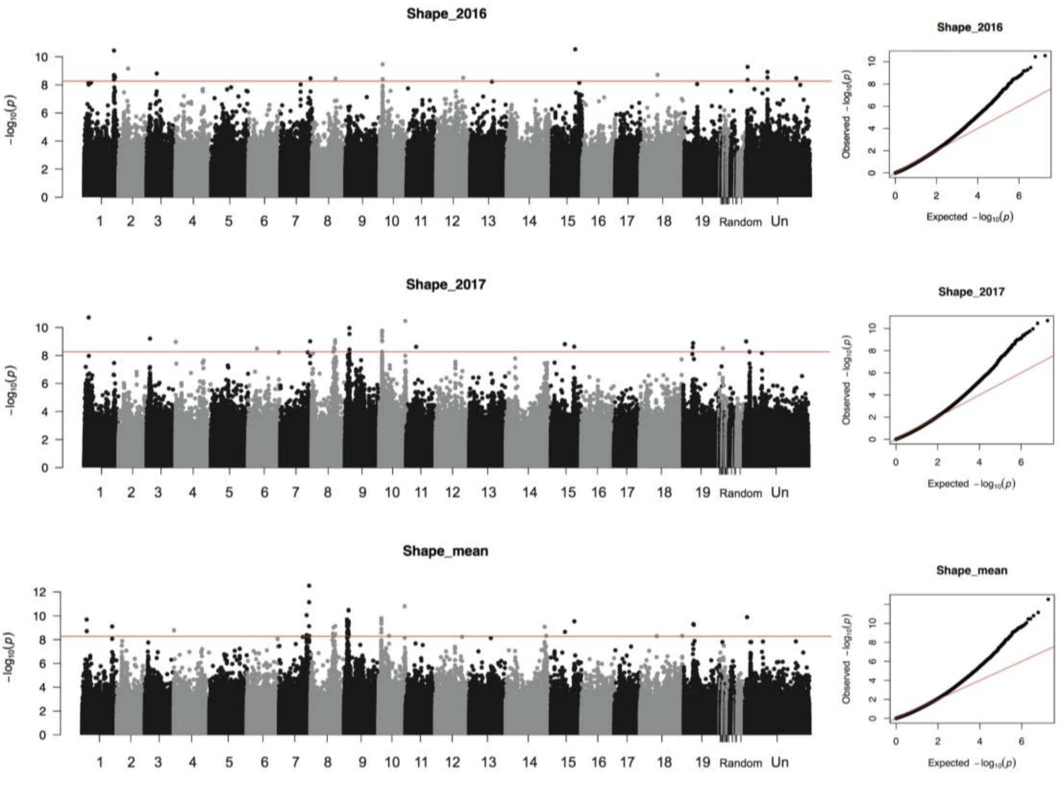

Supplementary Figure 8 GWAS of the grapevine berry shape trait collected in years 2016 and 2017 in available accessions using the compressed MLM. Mean berry shape trait is the average value of year 2016 and 2017. The genome-wide Bonferroni significance threshold is shown as a red line.

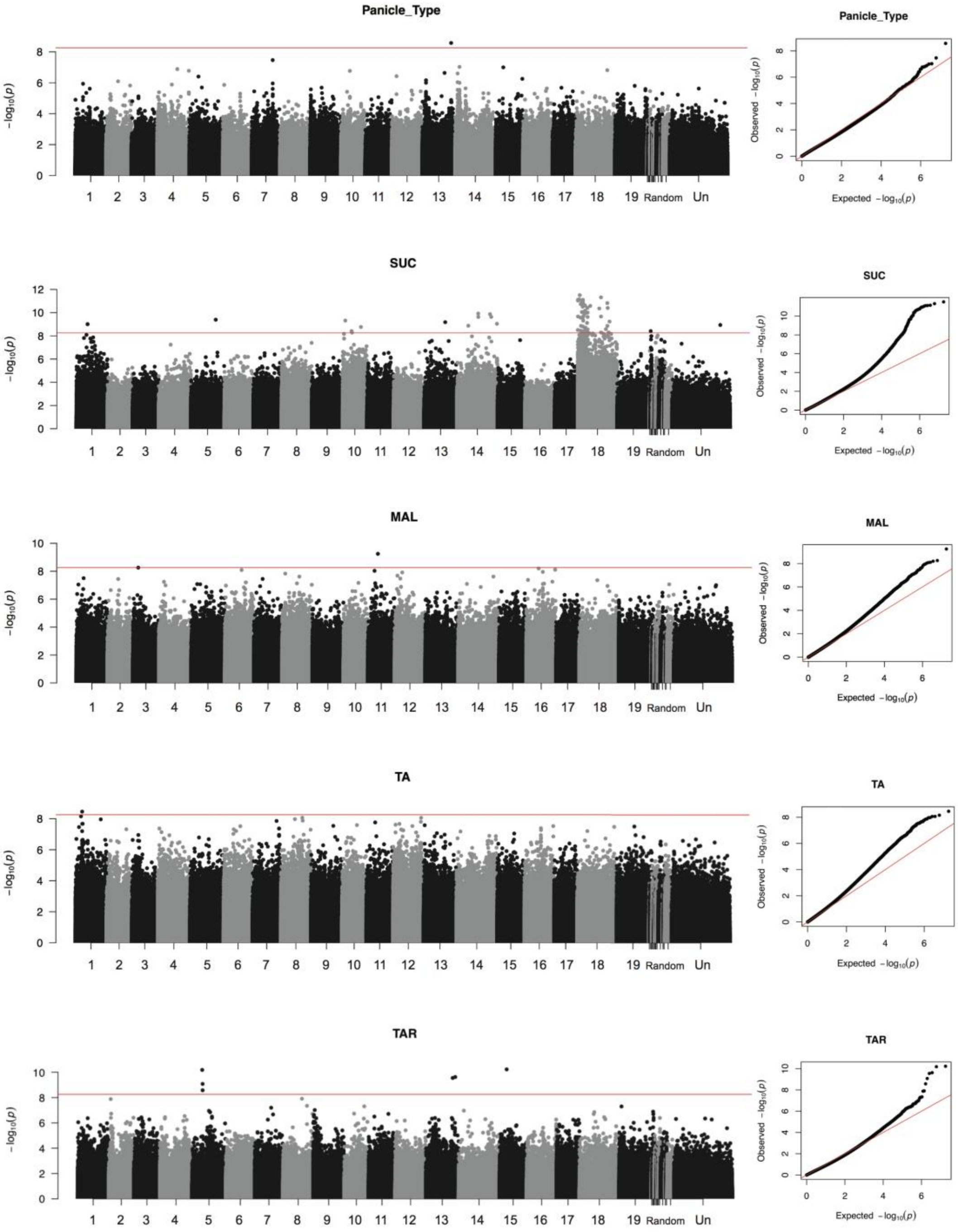

Supplementary Figure 9 GWAS of grapevine panicle type, SUC (berry sucrose content), MAL (berry malic acid content), TA (berry total acid content), and TAR (berry tartaric acid content) in available accessions using the compressed MLM. The genome-wide Bonferroni significance threshold is shown as a red line.

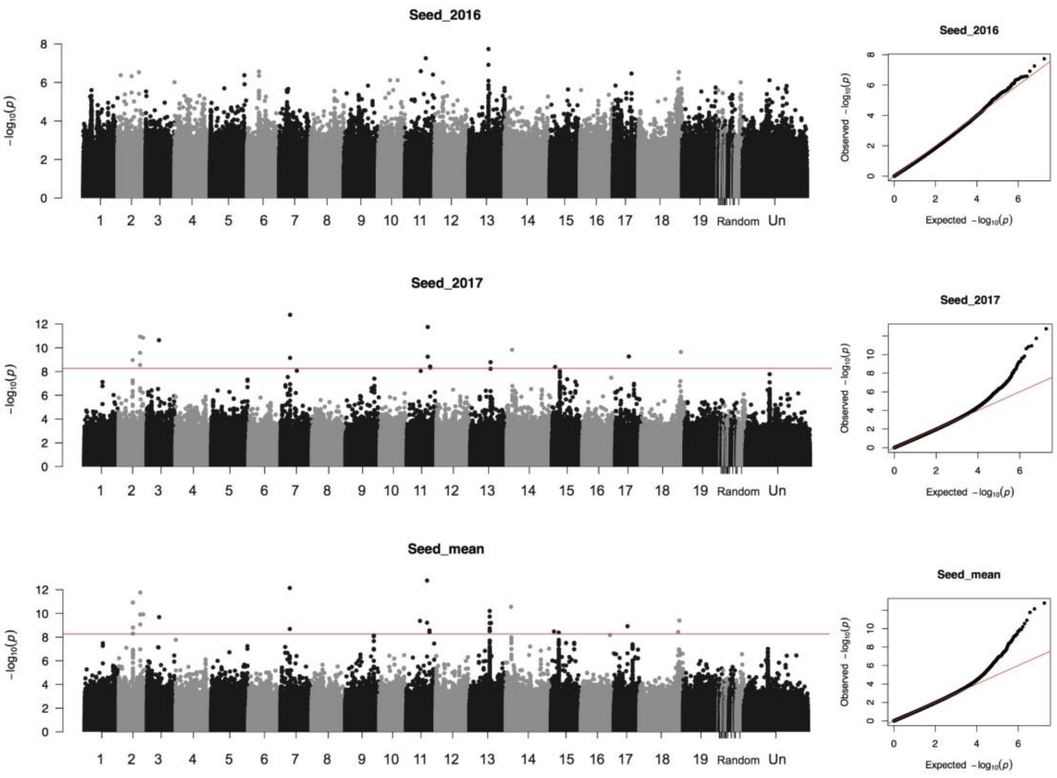

Supplementary Figure 10 GWAS of grapevine number of seeds in available accessions using the compressed MLM. The genome-wide Bonferroni significance threshold is shown as a red line. No significant signals were found in Seed-2016.

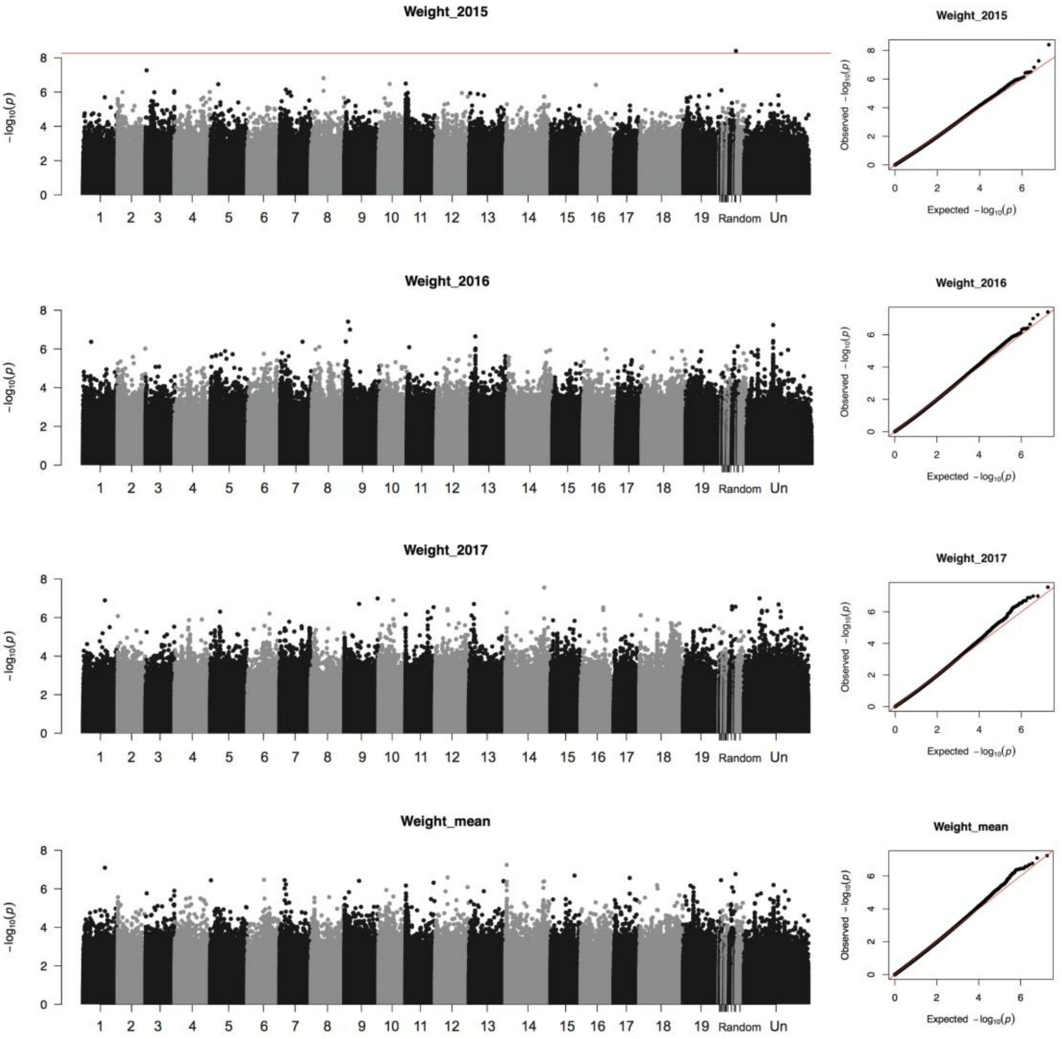

Supplementary Figure 11 GWAS of grapevine berry weight in available accessions using the compressed MLM. The genome-wide Bonferroni significance threshold is shown as a red line. No significant signals were found in weight 2016, 2017, and pooled mean.

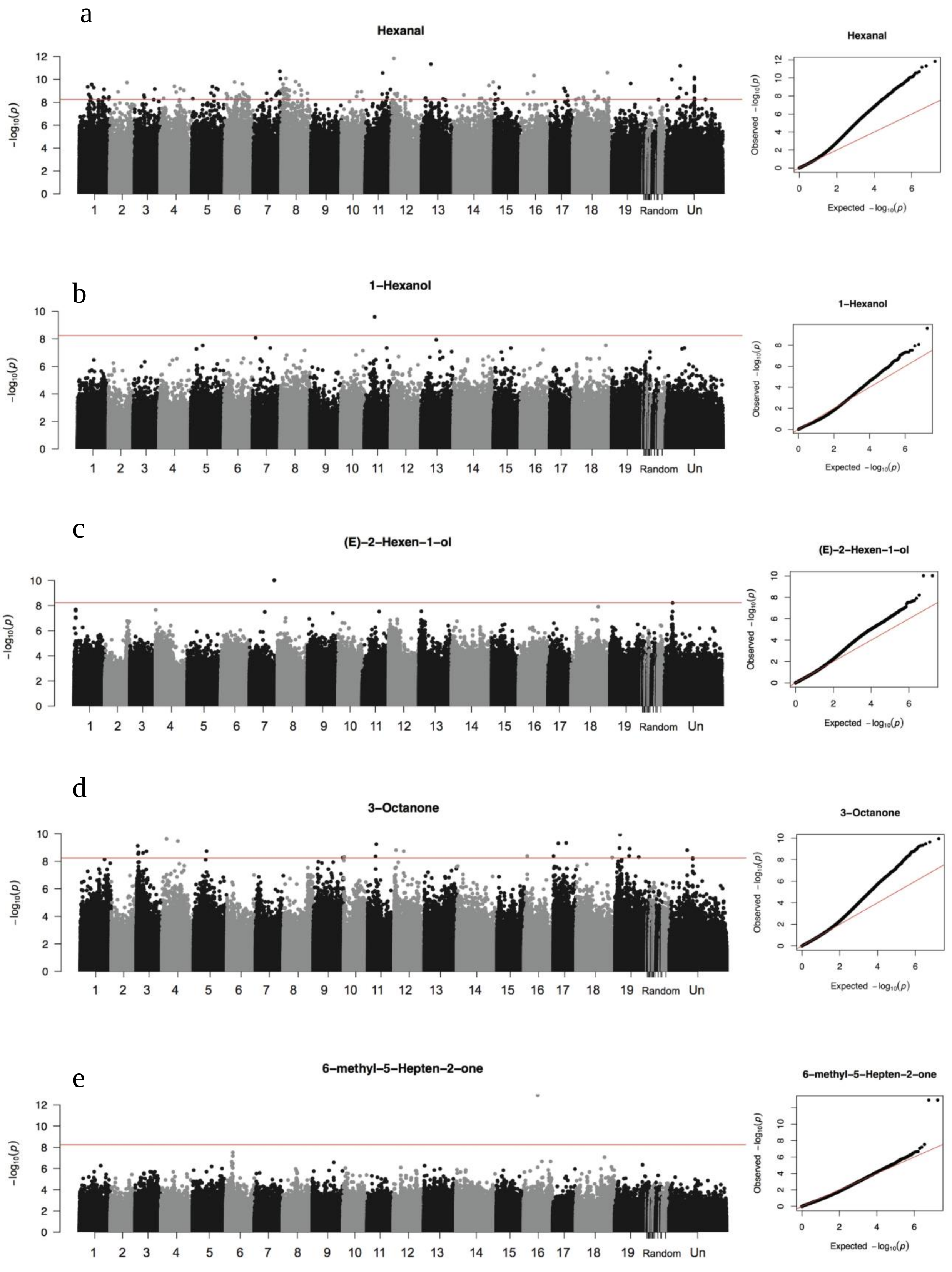

a
Hexanal
b
1−Hexanol
c
(E)−2−Hexen−1−ol
d
3−Octanone
e
6−methyl−5−Hepten−2−one
−log10(p)
1 2 3 4 5 6 7 8 9 10 11 12 13 14 15 16 17 18 19 Random Un
Observed −log10(p)
Expected −log10(p)

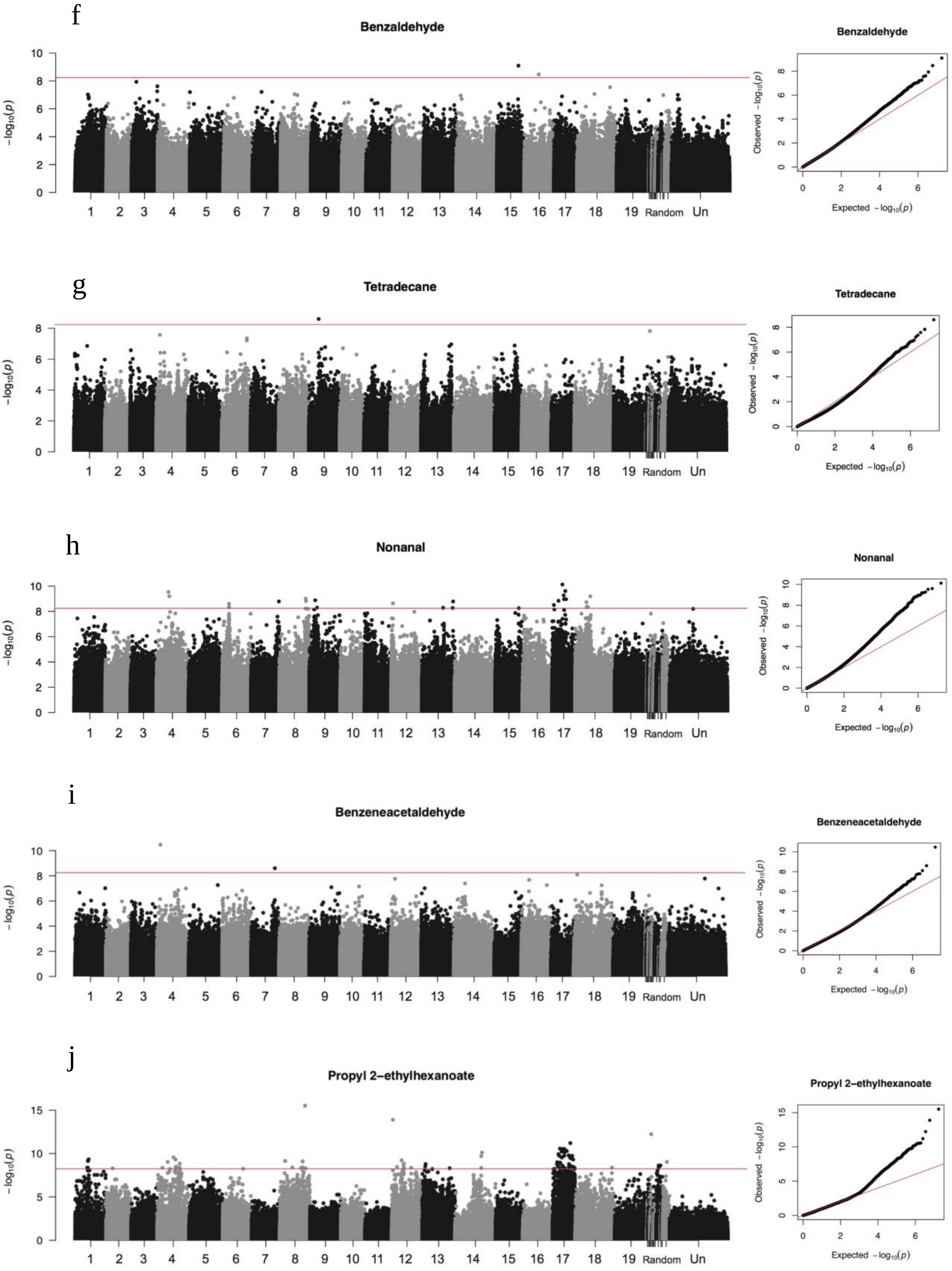
f
Benzaldehyde
g
Tetradecane
h
Nonanal
i
Benzeneacetaldehyde
j
Propyl 2−ethylhexanoate
$-\log_{10}(p)$
1 2 3 4 5 6 7 8 9 10 11 12 13 14 15 16 17 18 19 Random Un
Observed $-\log_{10}(p)$
Expected $-\log_{10}(p)$

k

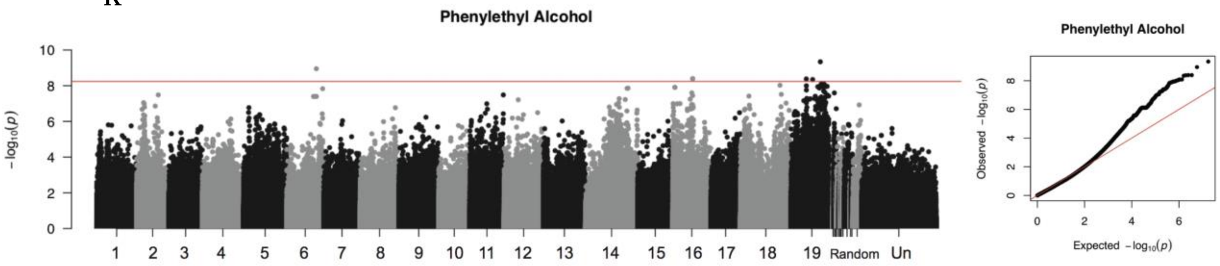

l

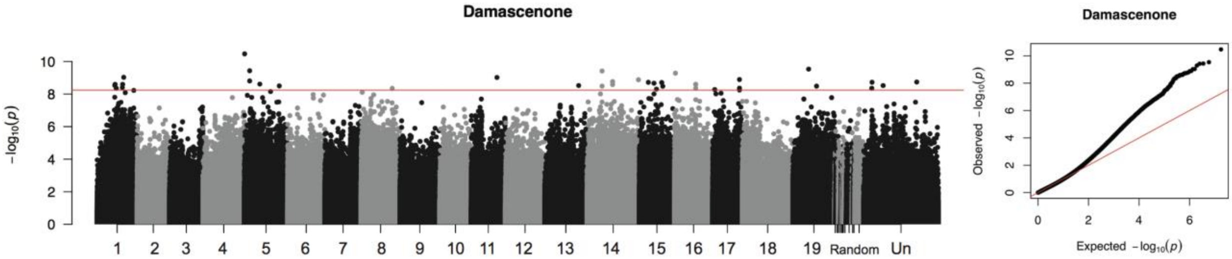

Supplementary Figure 12 GWAS of grapevine aroma content traits in available accessions using the compressed MLM. (a) hexanal; (b) 1-hexanol; (c) (E)-2-hexen-1-ol; (d) 3-octanone; (e) 6-mthyl-5-hepten-2-one; (f) benzaldehyde; (g) tetradecane; (h) nonanal; (i) benzeneacetaldehyde; (j) propyl 2-ethylhexanoate; (k) phenylethyl alcohol; (l) damascenone.

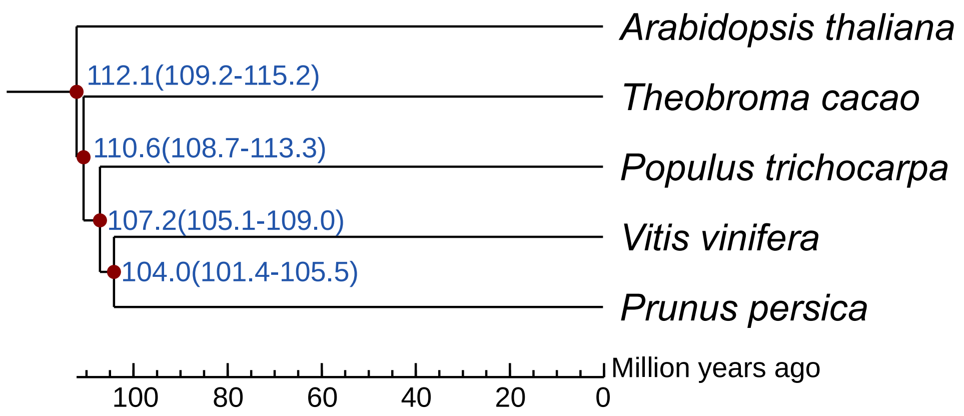

Supplementary Figure 13 The divergence times used for estimation of the neutral mutation rate of grapevine between *Prunus persica* and *Vitis vinifera*.
