# Supplementary material for: Whole-genome resequencing of 472 Vitis accessions for grapevine diversity and demographic history analyses
Source: Nat Commun. 2019 Mar 13;10:1190. doi: 10.1038/s41467-019-09135-8 (PMC6416300; doi:10.1038/s41467-019-09135-8)
Supplement: Supplementary file 3 — Description of Additional Supplementary Files [file 41467_2019_9135_MOESM3_ESM.docx]

**Description of Additional Supplementary Files**

**File Name:** Supplementary Data 1

**Description:** Summary of grapevine accessions.

**File Name:** Supplementary Data 2

**Description:** Summary of raw reads for *Vitis* accessions.

**File Name:** Supplementary Data 3

**Description:** Summary for selective sweep regions in CEU and WEU from CLR analysis.

**File Name:** Supplementary Data 4

**Description:** Summary for selective sweep regions during grapevine domestication from *F_ST_* and θπ.

**File Name:** Supplementary Data 5

**Description:** Summary of 24 grapevine phenotypes.

**File Name:** Supplementary Data 6

**Description:** Genes of genome-wide association signals for grapevine traits.

**File Name:** Supplementary Data 7

**Description:** Summary of the SNP signal detected by GWAS.
